# Manufacturing Uniform Cerebral Organoids for Neurological Disease Modeling and Drug Evaluation

**DOI:** 10.34133/bmr.0104

**Published:** 2024-11-06

**Authors:** Hyowon Hong, Yesl Jun, Sae-Bom Yoon, Seoyoon Park, Jaemeun Lee, Jeong Woon Jang, Hye Jin Nam, Heeyeong Cho

**Affiliations:** ^1^ Therapeutics & Biotechnology Division, Korea Research Institute of Chemical Technology, Daejeon, Republic of Korea.; ^2^ Medicinal Chemistry and Pharmacology, University of Science and Technology, Daejeon, Republic of Korea.

## Abstract

Human cerebral organoids are promising tools for investigating brain development and the pathogenesis underlying neurological disorders. To use organoids for drug effectiveness and safety screening, the organoids dispensed into each well must be prepared under precisely the same conditions as the cells. Despite decades of extensive research on approaches to improve organoid generation, various challenges remain, such as low yields and heterogeneity in size and differentiation both within and between batches. Here, we newly established uniform cerebral organoids (UCOs) derived from induced pluripotent stem cells by optimizing organoid size and performing real-time monitoring of telencephalic differentiation marker expression. These organoids exhibited morphological uniformity and consistent expression of *FOXG1* during telencephalic differentiation, with high productivity. Moreover, UCOs faithfully recapitulated early corticogenesis, concomitant with the establishment of neuroepithelial populations, cortical plate neurons, and glial cells. Furthermore, UCOs systematically developed neural networks and exhibited both excitatory and inhibitory electrophysiological signals when exposed to neurotransmission blockers. Neurodevelopmental disease models derived from UCOs manifested neurite outgrowth defects, which could be ameliorated with targeted drug treatment. We propose UCOs as an advanced platform with low organoid variations and high reproducibility for modeling both brain development and neurological diseases.

## Introduction

Human cerebral organoids are self-organizing neural tissues that manifest various features of cortical development, such as the spatiotemporal regulation of gene expression trajectories and differentiation into committed cell types [1,2]. These 3-dimensional (3D) models recapitulate the structural specificity of the developing cerebrum, including features such as apical lumens and neural buds, which are suggestive of the neural tube, as well as layered cortical neurons undergoing radial migration to the marginal zone of the cortex [1,2]. In addition, cerebral organoids generate specialized neurons and glial cells from neuroepithelial cells, following the developmental timeline observed in vivo. Similar to the anatomy and physiology of the human fetal brain, organoids demonstrate functional neurotransmission with mature synaptic structures [3,4]. Action potentials and network signals, which are susceptible to neurotransmission blockers, have been observed in cerebral organoids, indicating the presence of neural networking systems with functional synapses [5,6]. Therefore, cerebral organoids can serve as a compatible system for demonstrating and investigating the physiology of human cerebral development and the pathogenesis of neurodevelopmental disorders in vitro.

Current cerebral organoid models have predominantly been applied to qualitative investigations, with a primary focus on recapitulating in vivo conditions [7,8]. Numerous neurodevelopmental disease models derived from cerebral organoids have been established through genetic engineering or patient-derived induced pluripotent stem cells (iPSCs); thus, they mainly provide insight into the pathogenic characteristics and related molecular mechanisms observed in patients [9,10]. There is a continuously increasing demand for the use of 3D organoid models as a platform for high-throughput drug screening (HTS). However, there remain challenges owing to the lack of a standardized protocol to ensure consistent and uniform production of organoids with high quality and yield [11,12]. This is attributed to the inherent heterogeneity observed in cerebral organoids during differentiation and maturation from embryoid bodies (EBs) [13,14]. Without achieving intra-batch homogeneity and batch-to-batch reproducibility, organoid-based drug evaluations remain unreliable. To overcome this issue, the characteristics of organoids to be manufactured must be evaluated, and standardized culture methods for reproducible organoid production must be established.

In this study, we newly established a high-quality uniform cerebral organoid (UCO) model as a tool for drug screening that demonstrated homogeneity in both organoid size and telencephalic differentiation. To improve the uniformity of size and telencephalon-specific fate determination, organoids were generated by regulating the aggregation of iPSC colonies within microwells and implementing the Wnt inhibition process during neural induction. Additionally, we developed the reporter iPSC line that expresses mCherry proteins that are dependent on the expression of FOXG1, which is a key indicator of telencephalic differentiation. One month after organoid culture began, the UCOs exhibited low size variation, and a large proportion of UCOs showed high mCherry expression. Moreover, the UCOs followed a timeframe similar to that of fetal brain development, exhibiting various committed cell types and region-specific gene expression profiles. Subsequently, the UCOs showed more prominent neuroelectrical activity than the unstandardized organoid model, which was indicative of advanced neuronal functionality. Finally, we established a neurodevelopmental disease UCO model, which displayed pathogenic phenotypes that were analyzed using the high-content screening (HCS) system and restored by pharmaceutical intervention. Taken together, our findings suggest that the UCO is an ideal platform for targeted drug screening, leveraging high-throughput automatic image analysis, based on their advanced uniformity and high productivity.

## Materials and Methods

### iPSC and UCO culture

Human iPSCs (IMR90-4, WiCell) were cultured on Matrigel-coated culture dishes or 6-well plates with mTeSR1 plus media (100-0276, STEMCELL Technologies). Cells were passaged every 3 to 4 d using the Gentle Cell Dissociation Reagent (100-0485, STEMCELL Technologies) to dissociate iPSC colonies into small cell clumps. Organoids derived from spontaneous aggregation (SA) and single-cell aggregation in a 96-well ultra-low attached plate (96W) were produced as previously described [15]. UCO generation was based on the SA organoid method with modifications. When the iPSCs reached a confluence of 70% to 80%, the cells were detached from the culture dishes as clumped colonies using a low concentration of dispase (Invitrogen: 17105-041; 0.7 mg/ml) for 30 min in a 37 °C incubator. Suspended iPSC clumps were resuspended in mTeSR1 plus media with 10 μM Y-27632 and gently pipetted using a 5-ml serological pipette. Small pieces of iPSC clumps were spread into the 400, 600, and 1,000 μm of microwells (StemFIT 3D, MICROFIT) and cultured for 1 d to obtain EBs homogeneously. Subsequently, EBs were collected and cultured in 100-mm ultra-low attachment (ULA) dishes (4615, Corning) with mTeSR1 plus media for an additional day. Neural induction was started by replacing the neural induction media [Dulbecco’s modified Eagle’s medium/F12 (12634-010, Gibco) containing Glutamax (35050-061, Gibco), 20% KnockOut Serum replacement (10828-010, Gibco), 1% Eagle’s minimum essential medium–nonessential amino acid solution (MEM-MEAA; 11140-050, Gibco), 1% penicillin/streptomycin (15140-122, Gibco), and 0.1 mM β-mercaptoethanol] with dual SMAD inhibitors (10 μM dorsomorphin; P5499, Sigma and 10 μM SB431542; S4317, Sigma) for 3 d. On day 4, the EBs were transported to 6-well ULA plates, and 2.5 μM IWP-2 (HY13912, MedChem Express) was added to the neural induction media. From days 10 to 14, the media were changed daily to neural progenitor cell (NPC) expansion media [Neurobasal A medium (10888-022, Gibco) containing B27 supplement minus VitA (12587-010, Gibco), Glutamax, penicillin/streptomycin] supplemented with fibroblast growth factor 2 (FGF2) (3718-FB-01M, R&D Systems) and epidermal growth factor (EGF) (236-EG-01M, R&D Systems). On day 15, NPC expansion media were changed every other day until day 24. On day 25, media were changed to neuronal differentiation media, which were basally the same as the NPC expansion media, and growth factors were changed to brain-derived neurotrophic factor (BDNF; 248-BDB-01M, R&D Systems and NT-3;267-N3-025, R&D Systems). On day 43, the organoids were cultured by a basal medium by excluding the growth factors from the neuronal differentiation media, and the media were replenished every 3 d.

### Generation of reporter iPSC lines

To create the FOXG1-mCherry donor plasmid, we obtained all homology arms via polymerase chain reaction (PCR) from iPSC genomic DNA (gDNA) pools. The mCherry-LoxP-PGK-PuromycinR cassette-LoxP construct was generated and inserted into the pUC19 vector using NEBuilder HiFi DNA Assembly (E2621L, NEB) or overlap PCR (KME-101, TOYOBO). The DNA sequence of the mCherry-LoxP-PGK-PuromycinR cassette-LoxP was inserted between the left and right homology arms using overlap PCR or NEBuilder HiFi DNA Assembly in the pUC19 vector. The single-guide RNA (sgRNA) targeting the FOXG1 C-terminal locus (target: CAGGGATGTTAATGTATTAA) was constructed in the px330 vector (Addgene #42230). AAVS1-EGFP cells were generated using the px330-Cas9-AAVS1-T2 sgRNA (Addgene #72833) and the AAVS1-donor-T2A Puro-CAG-EGFP plasmid (Addgene #80945). Plasmids were introduced by electroporation (Neon, Invitrogen), and iPSCs were subjected to puromycin treatment (0.5 μg/ml) for 3 d starting from 3 to 4 d after transfection. Following puromycin selection, cells underwent single-colony selection. The RevitaCell Supplement (A2644501, Thermo) was used to improve cell viability during puromycin and single-cell selection. The FOXG1-mCherry and AAVS1-EGFP knock-in iPSCs were validated using junction PCR and Sanger sequencing. In addition, because FOXG1-mCherry iPSCs were generated heterozygously, validation of the other wild-type (WT) allele was performed using Sanger sequencing.

### gDNA extraction and PCR for genotyping

Cells were lysed at 60 °C for 15 min using a gDNA lysis buffer [40 mM tris-hydrochloride (pH 8.0), 1% Tween-20, 0.2 mM ethylenediaminetetraacetic acid, 20 mg/ml proteinase K, and 0.2% Nonidet P-40]. The lysates were then incubated at 98 °C for 5 min. For PCR amplification, 100 to 200 ng of gDNA were used as a template. All PCRs were performed using KOD Multi & Epi (KME-101, TOYOBO). The primer sequences used are listed in Table S1.

### Imaging of organoids

Bright-field and fluorescence live imaging of the organoids was performed using the EVOS XL Core system (Invitrogen, USA) and a Cytation C10 confocal microscope (Agilent Biotek, USA). For immunohistochemistry, the organoids were fixed in 4% paraformaldehyde (PFA) for 2 h, submerged in 30% buffered sucrose overnight for cryoprotection, and embedded in gelatin blocks [7.5% gelatin and 10% sucrose in phosphate-buffered saline (PBS)]. Subsequently, 20 μm of cryostat sections was placed on glass slides and hydrated with PBS for 10 min. Samples were treated with a blocking solution (10% normal goat serum/PBS) containing 0.1% Triton X-100 for 1 h at room temperature (RT), followed by serial labeling with primary and secondary antibodies. For immunocytochemistry, cells were fixed in 4% PFA for 7 to 10 min at RT and washed with PBS 3 times. Fixed cells were treated with a blocking solution with 0.05% Triton X-100 for 10 min at RT and stained by serial primary and secondary antibody treatment. Images were automatically analyzed using the Gen5 software (Agilent Biotek, USA). Antibodies used in this study are listed in Table S2.

### RNA preparation and real-time quantitative PCR

Total RNA was extracted using the Hybrid-R RNA prep kit (305101, GeneAll) according to the manufacturer’s instructions. Complementary DNA (cDNA) was synthesized from 200 ng of extracted RNA using the cDNA synthesis master mix (6290, LeGene Biosciences). Synthesized cDNA was mixed with primers and iQSYBR Green Supermix (1708882, Bio-Rad) to perform real-time quantitative PCR (qPCR) on the CFX Connect Real-Time System (Bio-Rad, USA). Relative gene expression was analyzed using the ΔΔCt method using the following primer pairs: FOXG1 (forward: AGAAGAACGGCAAGTACGAGA, reverse: TGTTGAGGGACAGATTGTGGC) and GAPDH (forward: GTCTCCTCTGACTTCAACAGCG, reverse: ACCACCCTGTTGCTGTAGCCAA).

### Total RNA sequencing

A library was independently prepared with 0.5 μg of total RNA samples from iPSCs (IMR90-4) and SA organoids/UCOs at 3 time points using the Illumina TruSeq Stranded Total RNA Library Prep Gold Kit (20020599, Illumina Inc.). Library quantification was conducted using the KAPA Library Quantification kits for Illumina Sequencing platforms following the qPCR Quantification Protocol Guide (KAPA BIOSYSTEMS). Qualification was performed using the TapeStation D1000 ScreenTape (Agilent Technologies, USA). Indexed libraries were subsequently submitted to Illumina NovaSeq (Illumina Inc., USA), and paired-end sequencing (2 × 100 base pairs) was conducted by Macrogen Incorporated. The reference genome sequence (hg19) and annotation data were downloaded from the University of California, Santa Cruz table browser (http://genome.uscs.edu). The StringTie software (v2.1.3b) was used to assemble aligned reads into transcripts and estimate their abundance, where the relative abundance estimates were provided as read counts for the transcripts and genes expressed in each sample. The read counts were normalized and transformed by applying variance stabilizing transformation (VST) using DESeq2 version 1.40.2 [16]. The VST-applied counts were used for all subsequent analyses. Pearson’s correlation between samples was calculated using the rcorr() function of Hmisc version 5.1-1, and the result was visualized using ComplexHeatmap version 2.16.0 [17]. Principal components analysis (PCA) was conducted using the prcomp() function of Stats version 4.3.2 and visualized using ggplot2 version 3.4.4.

### BrainSpan RNA sequencing database analysis and transition mapping

The BrainSpan transcriptome data of the human brain were used as an in vivo reference for the analysis. For stage- and region-specific identity analyses, the data from the organoids and the in vivo reference were combined and processed as previously reported [18]. For the BrainSpan dataset, CEL files containing raw microarray data were downloaded from Gene Expression Omnibus (GEO; accession number: GSE25219). Background subtraction, quantile normalization, and summarization of the raw data were performed using the rma() function, and the expression matrix containing the expression values of 17,410 genes for every sample was extracted using the exprs() function of oligo version 1.64.1. To extract region-specific markers for the heatmap, samples at approximately 13 to 19 post-conception weeks (PCW) were selected and grouped into 6 groups by region [dorsal telencephalon for the primary auditory cortex (A1C), dorsolateral prefrontal cortex (DFC), posterior inferior parietal cortex (IPC), inferior temporal cortex (ITC), primary motor cortex (M1C), and primary somatosensory cortex (S1C), medial prefrontal cortex (MFC), orbitofrontal cortex (OFC), superior temporal cortex (STC), and primary visual cortex (V1C); ventral telencephalon for the striatum (STR); thalamus for the midbrain (MD); hippocampus (HIP); cerebellum (CERB); amygdala (AMY)]. Differentially expressed gene (DEG) analyses were used to compare samples that did and did not belong to a group using limma version 3.56.2. The top 50 significant DEGs (according to adjusted *P* values < 0.05 using the Benjamini–Hochberg procedure) based on fold change were selected as markers of the region group. For transition mapping (TMAP), we performed rank–rank hypergeometric overlap (RRHO) analysis between the BrainSpan dataset and the transcriptomic organoid data. The BrainSpan samples were grouped by age into 11 stages. To obtain the ranks of the genes in stages 2 to 11, DEG analyses were performed on the BrainSpan samples by comparing each stage with stage 1 (5 to 7 PCW) using limma. The organoid samples were grouped into 6 groups by culture condition and day. To obtain the ranks of the genes in each group, DEG analyses were conducted to compare organoid samples in the group with iPSC samples. For the combination of BrainSpan stages and organoid groups, RRHO analysis was performed using the RRHO() function of RRHO version 1.40.0. Input values were calculated using the following formula:

$$\begin{array}{c} \text{Input values}=-{\mathrm{Log}}_{10}\left(\text{adjusted}\;P\;\text{value}\;\mathrm{by}\;\text{Benjamini}\right.\\ \;-\left.\text{Hochberg procedure}\right)\cdot \text{sgn}\;\left(\text{Fold change}\right) \end{array}$$

The step size for the overlap test was 100. The heatmap and RRHO map were generated using ComplexHeatmap and ggplot2.

### Electrophysiological recording using a multi-electrode array

To record neuroelectrical signals from organoids, multi-electrode array (MEA) 24-well plates (Axion Biosystems, USA) were precoated with 0.1% polyethylenimine solution and 10 μg/ml laminin. After washing and drying the coating solution, the 100-d-old organoids were plated onto the wells, and the entire electrode area was covered and incubated at 37 °C for 30 min. The culture media were then carefully added to each well and changed every other day. Recordings were performed using the MaestroEDGE MEA system and AxIS Software Spontaneous Neural Configuration (Axion Biosystems, USA). Spikes were detected using the AxIS software with the same setting values as those used in a previous study [5]. The plates were first rested for 30 to 60 min inside the Maestro device, and 10 min of data were recorded. The minimum spike rate for an active electrode was set to 5 spikes/min, and bursts, defined as clusters of spikes, were identified using an interspike interval (ISI) threshold in which a minimum of 5 spikes with a maximum ISI of 100 ms was required. Network bursts required a minimum of 50 spikes with the same ISI, with a minimum of 35% participating electrodes in the well. The synchrony index was estimated using a cross-correlogram synchrony window of 20 ms. 2D neurons, singularly dissociated from organoids, were seeded onto the electrode area in each well at a density of approximately 0.5 × 10^5^ cells and incubated for 2 to 5 weeks. For drug treatment, baseline recordings were performed immediately before and 10 min after drug administration. The following drugs were used for the treatment: 1 μM tetrodotoxin (TTX; 1078, Tocris), 1 to 30 μM baclofen (G013, Sigma), 32 μM 1(S),9(R)-(-)-bicuculline methiodide (14343, Sigma), 1 to 3 μM MK801 (M107, Sigma), and 1 μM rotenone (R8875, Sigma). The data were analyzed using the AxIS navigator software and the Neural Metric Tool, which is a standalone tool provided by the manufacturer (Axion Biosystems).

### Single-cell dissociation of organoids

To examine organoid dissociation, the papain dissociation kit (Worthington Biochemical Corporation, USA) was used according to the manufacturer’s instructions. Briefly, all kit solutions were oxygenated for 5 min with 95% oxygen and 5% carbon dioxide, and the organoids were dissected into small pieces in an oxygenated papain solution. The dissected organoids with papain solution were incubated at 37 °C while gently inverting for 1.5 h. Subsequently, gentle trituration was performed to increase the yield, and papain was inactivated using an albumin–ovomucoid inhibitor solution.

### Neurite outgrowth assay and drug treatment

To assess the neurite lengths in the 2D culture, the early neurons were immunostained using anti-TUBB3 antibodies, and the neurites were analyzed via fluorescence imaging and image processing using a Cytation C10 confocal microscope and the ImageJ software (National Institutes of Health, USA). To assess them in the 3D organoids, enhanced green fluorescent protein (EGFP)-expressed WT- and Rett syndrome (RTT)-UCOs at day 40 were attached to 24-well plates primarily coated with 1% (v/v) Matrigel (354230, Corning) and 10 μg/ml laminin (L2020, Sigma). The following drugs were used for the treatment: 0.1 μM nocodazole (M1404, Sigma), 0.03 to 3 μM trofinetide (HY-16757, MedChemExpress), 0.03 to 3 μM blarcamesine hydrochloride (HY-101864, MedChemExpress), and 25 ng/ml BDNF. Neurite outgrowth was observed daily using a Cytation C10 confocal microscope, and the acquired raw images were processed using the Gen5 software from the Cytation C10 image system. Areas of outgrown neurites labeled by EGFP in the processed images were quantified using the ImageJ software.

### Statistics

Data analysis was conducted using the Prism 8 software (GraphPad Software Inc., USA). Statistical significance was evaluated using 2-tailed unpaired Student’s *t* tests to compare the SA groups with other groups and UCOs.

## Results

### Aggregation of iPSC clumps in the microwell contributes to the size uniformity of the cerebral organoid

**Fig. 1.**
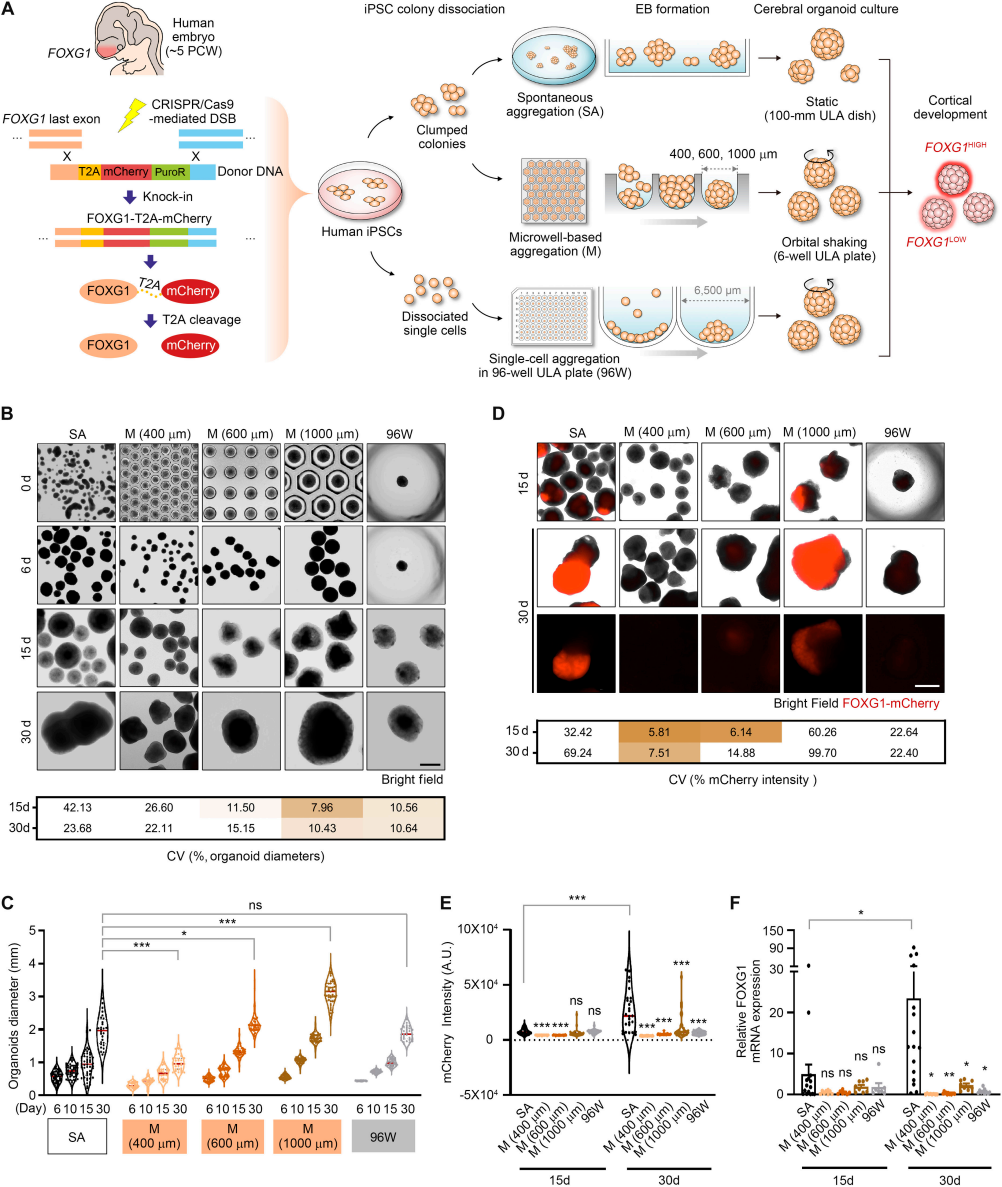
Microwell-based aggregation of iPSC clumps makes organoids amenable to uniform growth. (A) Schematic diagrams representing the overall processes of EB formation and cerebral organoid culture using FOXG1-mCherry-expressed iPSCs in culture dishes, microwells, and 96-well ULA plates. DNA cassette for mCherry expression was inserted into the 3′ end of the last *FOXG1* exon using the CRISPR/Cas9-mediated knock-in system. (B) Bright-field images showing EBs and organoids from the indicated conditions at each time point. Table showing the CV (%) values of the size of organoids in each group at days 15 and 30. The CV represents the percentage of the standard deviation divided by the mean value. (C) Dot plot showing the diameters of organoids in the indicated conditions. (D) Live images showing FOXG1-mCherry-expressed organoids of the indicated groups at days 15 and 30. The standard exposure time of the laser for mCherry fluorescence was 400 ms, and the low exposure time was 100 ms. Table showing the CV (%) values of the mCherry intensity of organoids in each group at days 15 and 30. The CV represents the percentage of the standard deviation divided by the mean value. (E and F) Dot plots showing the mCherry intensity (E) and relative *FOXG1* mRNA expression, normalized by its expression in iPSCs (F), in the organoids of each group at days 15 and 30. All data are expressed as mean ± standard error of the mean (SEM), and significance of each group was calculated by comparing with the SA group. *P < 0.05, **P < 0.01, *** *P* < 0.001, and ns: not significant. Scale bars, 1 mm.

To achieve morphological and phenotypical consistency of the cerebral organoids, we used various platforms to generate uniformly sized EBs from iPSCs and compared their size uniformity and differentiation potency during early cortical development (Fig. 1A). To investigate whether EBs accurately undergo telencephalic differentiation throughout the organoid culture, we established a reporter system in iPSCs to invasively detect the expression of *FOXG1*, a major marker of telencephalon, by using clustered regularly interspaced short palindromic repeats (CRISPR)/CRISPR-associated protein 9 (Cas9)-based knock-in system (Fig. 1A and Fig. S1A). The reporter system enables the straightforward detection of FOXG1 expression by observing the mCherry fluorescence signal.

We first assembled the EBs from the iPSC colonies, which were enzymatically detached from a culture dish and spontaneously aggregated into 3D spheroids over a few hours (SA in Fig. 1A). These EBs were cultured following the methods of Paşca et al. [2] (Fig. S1B). To improve the size uniformity of the EBs, we seeded small clumps of iPSC colonies in the concave-shaped microwells with varying diameters [400, 600, and 1,000 μm; microwell-based aggregation (M) in Fig. 1A]. We allowed iPSC colonies of various sizes to completely fill the microwell structure. Those that were not trapped in the well were washed out, which resulted in the formation of a spherical EB within each microwell. Thus, the size of the EBs was determined by the dimensions of the microwells. Intact EBs that formed in the microwells were transferred to 100-mm ULA dishes, and neuroectodermal differentiation was started in the same way as in the SA method (Fig. S1B). Most previous studies have shown uniform EB formation by controlling the number of seeded cells in 96-well U- or V-bottom plates [15,19]. To enable comparisons with a previously published single-cell aggregation method, in one set of experiments, we dissociated iPSC colonies into single cells and aggregated EBs (0.9 × 10^4^ cells/well) in the 96-well ULA U-bottom plate (96W; single-cell aggregation; Fig. 1A) according to the methods of Xiang et al. [15] (Fig. S1B). For dynamic cell culture after generating size-controlled EBs, the EBs from both M and 96W groups were transferred to 6-well ULA plates and placed on an orbital shaker during neural induction. Throughout EB formation and neuroectodermal differentiation, we compared the size and mCherry fluorescence intensity of organoids generated using the SA, M, and 96W methods (Fig. 1B to F).

Because of the differences in the initial sizes of the iPSC colony clusters, the organoid sizes in the SA group exhibited substantial heterogeneity during neural induction. This heterogeneity persisted until day 30 when neural progenitors had actively expanded and started to produce early neurons (Fig. 1B and C). However, the organoids generated using the M and 96W methods showed superior size uniformity than that of SA during early brain development (Fig. 1B and C). Specifically, the coefficient of variation (CV) values remained below 20% in organoids generated from the 600- and 1,000-μm microwells and the 96W method (Fig. 1B, table below). In line with previous studies using the 96W method, which reported that by day 10, EB sizes range from 600 to 800 μm [19,20], our EBs attained similar diameters on day 10 when cultured using either the 600-μm microwells or the 96W method (Fig. 1B and C). We also observed that microwell-based EB formation reduced the size difference, which inversely correlated with the increase in well diameter (Fig. 1B, table below). Moreover, the average size of organoids increased over time in correlation with the enlargement of well diameters, and the EBs from the 1,000-μm microwells grew to approximately 3 mm in diameter by day 30. These findings indicate that the size uniformity of EBs is sustained throughout the homogeneous aggregation process of iPSC clumps in 1,000-μm microwells at the onset of organoid culture.

As iPSCs differentiated into cerebral organoids, distinct variations in FOXG1-mCherry fluorescence intensity were observed for the various methods at days 15 and 30 (Fig. 1D and E). We observed a substantial increase in mCherry signals at day 30 in the organoids derived from SA. However, there was notable intra-batch variation (Fig. 1D, table below, and E). To validate the correlation between mCherry intensity and *FOXG1* mRNA expression, we performed quantitative PCR analysis and confirmed that the data were consistent with the respective mCherry intensities (Fig. 1F). Notably, the mCherry intensity of SA organoids at day 30 was positively correlated with organoid size, which indicated that the starting size of the spheroid is important for telencephalic differentiation (Fig. S1C). Indeed, both the 600-μm microwell and 96W organoids exhibited markedly low mCherry intensity and *FOXG1* expression at days 15 and 30 (Fig. 1D to F).

We also found that the 1,000-μm microwell organoids exhibited lower overall mCherry intensity and *FOXG1* expression than those observed in the SA group, although some organoids showed high mCherry intensity at day 30 (Fig. 1D to F). Furthermore, generating larger EBs by seeding 2 × 10^4^ cells in each well of the 96-well ULA plate did not enhance mCherry intensity or *FOXG1* expression, which suggested that the dissociation of iPSC colonies into single cells had a negative effect on the differentiation potency of the stem cells (Fig. S1D to G). Therefore, we selected the 1,000-μm microwell method as the ideal culture condition for obtaining uniformly sized organoids based on the low CV values for organoid size during early neuroectoderm differentiation. However, this method was not sufficient to consistently induce telencephalon-specific fate determination.

### Wnt inhibition during neural induction efficiently guides telencephalon-specific fate determination and synchronizes organoid growth

**Fig. 2.**
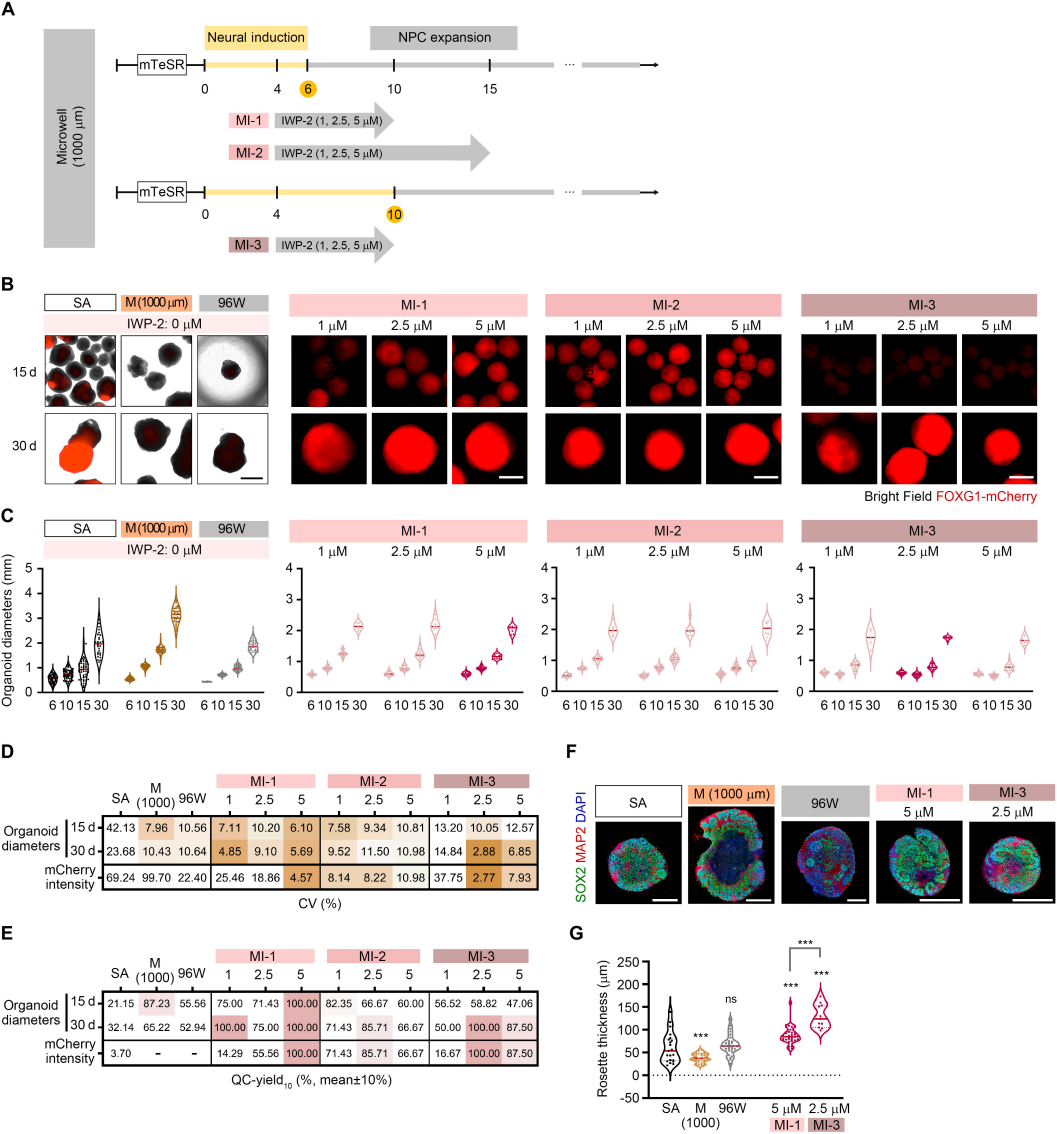
Wnt inhibition stimulates telencephalic differentiation and synchronizes organoid growth. (A) Schematic diagrams of the timeline and protocol for Wnt inhibition during neural induction following IWP-2 treatment at 3 doses (1, 2.5, and 5 μM) and various time points. Three types of protocols (M1-1, M1-2, and M1-3) were based on the 1,000-μm microwell culture condition described in Fig. 1A and Fig. S1B. (B) Live images presenting bright-field and FOXG1-mCherry fluorescence signals of organoids in each group at days 15 and 30. The exposure time of the laser in MI groups was 400 ms. (C) Dot plots showing the size of organoids in all groups at the indicated time points. (D and E) Tables showing the CV values (D) and QC-yield_10_ (%) (E) of organoid size and mCherry intensity (day 30) in the indicated conditions. The QC-yield_10_ was calculated as the percentage of the number of organoids within ±10% of the average values of all organoids. (F) Immunofluorescence images showing a cross-section of organoids stained with anti-SOX2 and anti-MAP2 antibodies at day 30. The nucleus was marked with 4′,6-diamidino-2-phenylindole (DAPI). (G) Dot plot showing the rosette thickness of organoids in the indicated groups. All quantitative data are expressed as mean ± SEM, and significance of each group was calculated by comparing with the SA group. *P < 0.05, **P < 0.01, ***P < 0.001, and ns: not significant. Scale bars, 1 mm.

To guide the EBs generated from iPSC clumps into the telencephalon, we applied Wnt inhibition during neural induction (Fig. 2A). The canonical Wnt signaling pathway is crucial for neural patterning during the early developmental stage, and telencephalic forebrain development is stimulated by both Wnt inhibition and bone morphogenetic protein (BMP)/transforming growth factor-β (TGF-β) inhibition [21,22]. Several guided cerebral organoid protocols use Wnt inhibitors, such as IWP-2 or XAV939, in combination with BMP/TGF-β inhibitors for driving a rostral–dorsal pallial fate during neural induction [15,20]. The SA method used in our study is a guided protocol used for cerebral organoid development that incorporates specific growth factors to enhance neuronal differentiation and maturation, which improves cortical lamination and synaptogenesis [2], but does not include the Wnt inhibition process. We hypothesized that omission of the Wnt inhibition process would lead to heterogeneity in fate determination among EBs.

Indeed, the EBs of the SA group showed large differences in sizes and mCherry intensity across various time points during the organoid culture process (Fig. 1C and E and Fig. S2A to F). Transferring the EBs to an orbital shaking condition did not improve EB size uniformity or mCherry expression (Fig. S2A to F). However, treatment with IWP-2, an inhibitor of the canonical Wnt pathway, in the SA group during neural induction markedly stimulated mCherry expression and reduced signal heterogeneity (Fig. S2A to F). Although Wnt inhibition failed to standardize EB sizes in the SA group (>20% CV), it was sufficient to trigger high levels of mCherry/*FOXG1* expression. The addition of the shaking process further elevated *FOXG1* expression beyond the saturation point of mCherry fluorescence intensity (Fig. S2A, C, and D). These results indicated that Wnt inhibition not only facilitates the up-regulation of FOXG1 expression, which is crucial for telencephalic fate determination during neural induction, but also enhances the uniformity of outcomes within the same batch.

Although *FOXG1* expression was triggered by Wnt inhibition, the size variation persisted because of the different onset sizes of EB aggregation in the SA group (Fig. S2E). We hypothesized that the application of a Wnt inhibitor to uniformly sized organoids generated from microwells would stimulate high levels of mCherry/FOXG1 expression and ultimately generate cerebral organoids with homogeneous size and differentiation. To test this hypothesis, we administered IWP-2 to the 1,000-μm M group, which previously showed homogeneous size and lower mCherry expression than the SA method (Fig. 1). We optimized the dosage and treatment timeline to ensure efficient and homogeneous FOXG1 induction. This optimization process involved comparing 3 different treatment conditions (i.e., Microwell-IWP-2 groups; MI-1, MI-2, and MI-3) with varying doses (i.e., 1, 2.5, and 5 μM) of IWP-2, as illustrated in Fig. 2A: (a) MI-1 represents the treatment of IWP-2 at the indicated doses for 10 d; (b) MI-2 is IWP-2 treatment for 15 d; and (c) M1-3 is IWP-2 treatment for 10 d, but neural induction was maintained during these periods. Both mCherry intensity and *FOXG1* expression were highly increased by Wnt inhibition in all MI groups (Fig. 2B and Fig. S2H and I). In particular, the 5 μM (IWP-2) group of MI-1 and the 2.5 μM (IWP-2) group of MI-3 showed minimal variability, with CV values below 5% (Fig. 2D). Unexpectedly, most MI groups showed greater size uniformity of organoids during the 30 d of cerebral organoid culture than the M group without IWP-2 (Fig. 2C and D). These findings suggest that Wnt inhibition synchronized the growth rate of organoids when their starting sizes were homogeneously regulated.

To determine the most favorable condition for producing uniform organoids, we calculated % yield as the percentage of the number of organoids within ±10% of the average diameter value of all organoids (Fig. 2E and Fig. S2G). We used this measure of quality-checked (QC) organoids as QC-yield_10_ to define the standard for organoid uniformity in each condition. The QC-yield_10_ for mCherry intensity was calculated as the proportion of organoid numbers within ±10% of the average intensity value exhibiting above the mean values of the SA group [approximately 2.6 × 10^4^ (A.U., artificial unit); laser exposure time: 400 ms]. We confirmed that all 30-d organoids in the 5 μM (IWP-2) group of MI-1 and the 2.5 μM (IWP-2) group of MI-3 met the QC-yield_10_ criteria for size and mCherry intensity. These results indicated that these 2 conditions were optimal for producing a highly UCO culture with homogeneous size and telencephalic differentiation. Next, we analyzed the neural rosette thickness of the organoids for each condition; the neural rosette comprises radially arranged columnar cells that express neuroepithelial cell-specific genes in the neural tube (Fig. 2F and G and Fig. S2J and K). We found that the 2.5 μM (IWP-2) group of MI-3 showed higher rosette thickness than the 5 μM (IWP-2) group of MI-1, which indicated that this particular condition adequately supports the expansion of neuroepithelial cells and the subsequent generation of their progenies, such as neurons, astrocytes, and oligodendrocytes, as neuronal differentiation progresses. Therefore, we propose that the 2.5 μM (IWP-2) condition of MI-3 represents the optimal protocol for producing high-quality cerebral organoids. Hereafter, we designate the organoids generated from this protocol as UCOs.

In addition to uniformity, we estimated the productivity of UCOs generated from equivalent numbers of iPSCs by comparing the SA and UCO groups (Table S3). From one plate of iPSCs, the SA and UCO groups generated approximately 250 and 290 EBs, respectively, but lost 11.9% to 24.0% of the EBs because of extremely small EB sizes or spontaneous apoptotic/necrotic cell death. By day 30, following the QC-yield_10_ criteria (mean ± 10%), we obtained approximately 4.6 qualified organoids in the SA group and 260 in the UCO group, with productivity rates of 1.8% and 88.1%, respectively. Even with QC-yield_30_ (i.e., estimated at a mean of ±30%), the productivity of the SA group increased to only 7.4%. These results suggested that the UCO method represents a cost-effective approach for mass production of organoids with high quality and uniformity.

### UCOs recapitulate in vivo human cortical brain development

**Fig. 3.**
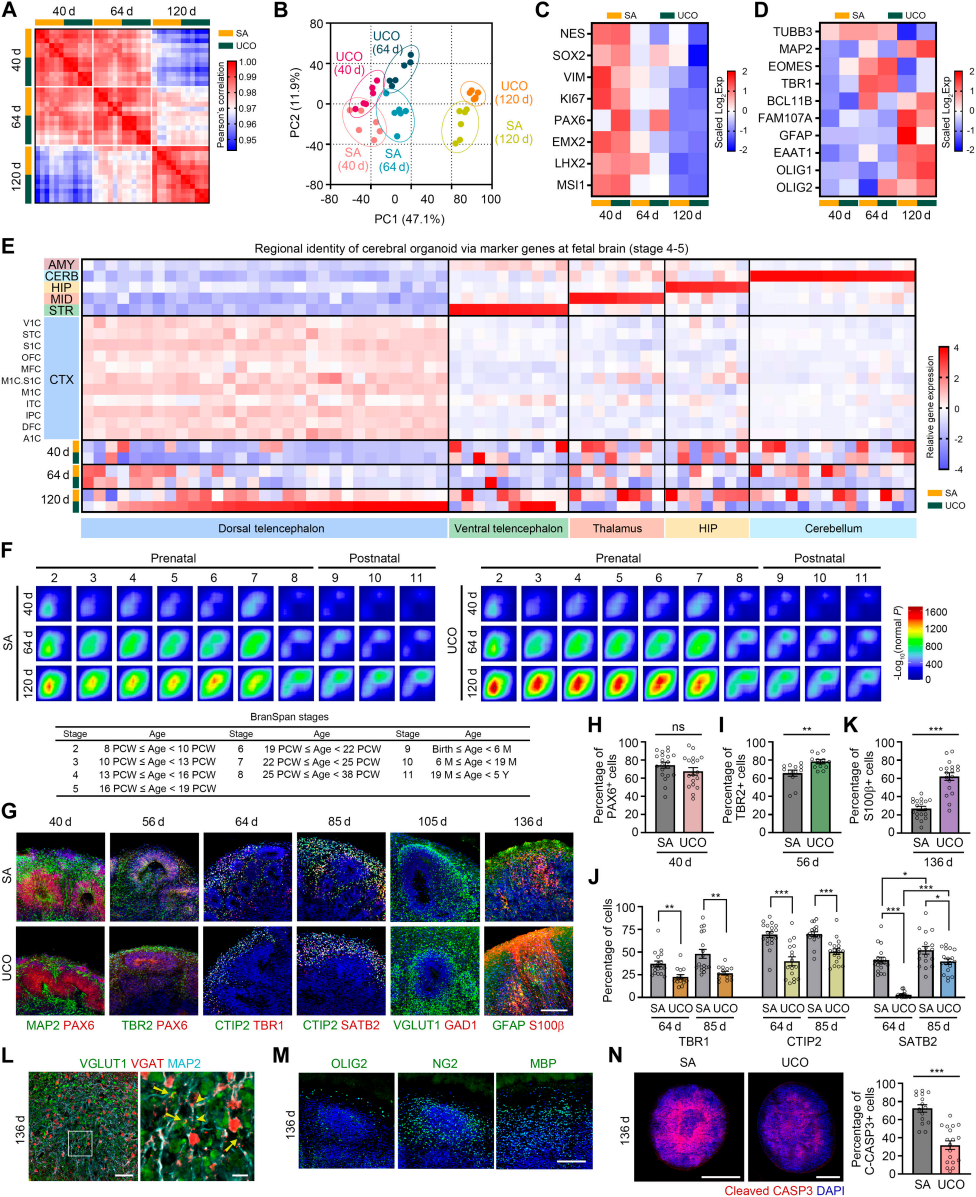
UCOs recapitulate cortical development with various types of committed cells, similar to the human fetal brain. (A and B) Correlation matrix (A) and PCA plot (B) showing the correlation between samples of UCOs and SA organoids at days 40, 64, and 120 in the total transcriptome analysis. (C and D) Heatmaps showing the expression of NPC (C) and early neuron-specific (D) genes in UCOs and SA organoids at each time point. The bar colors indicate the scaled logarithm based on 2 of normalized expression (i.e., scaled Log_2_Exp). (E) Heatmap showing the relative expression of region-specific markers (*x* axis) of the fetal brain during stages 4 to 5 (13 to 19 PCW) in the in vivo dissected region and the in vitro UCO/SA organoid samples (*y* axis). The color of the heatmap indicates the gene-wise scaled relative expression of the markers. CTX, cortex; A1C, primary auditory cortex; DFC, dorsolateral prefrontal cortex; IPC, posterior inferior parietal cortex; ITC, inferior temporal cortex; M1C, primary motor cortex; S1C, primary somatosensory cortex; MFC, medial prefrontal cortex; OFC, orbitofrontal cortex; STC, superior temporal cortex; V1C, primary visual cortex; STR, striatum; MID, midbrain; HIP, hippocampus; CERB, cerebellum; AMY, amygdala. (F) RRHO maps comparing the transition between in vivo developmental stages (stages 2 to 11 compared with stage 1) to UCOs/SA organoids (normalized to iPSCs). The bar colors signify the negative logarithm based on 10 of *P* values. The table shows the BrainSpan stages and their corresponding ages. PCW, post-conception weeks; M, months; Y, years. (G) Immunofluorescence images showing the edge regions of the UCOs/SA organoids at the indicated time points with staining for markers of NPCs (PAX6), IPs (TBR2), cortical layers (TBR1, CITP2, and SATB2), and glial cells (GFAP and S100β). The nucleus was stained with DAPI. (H to K) Bar graphs showing the percentages of cell populations with positive signals of indicated markers in the organoids at different time points. (L) Immunofluorescence images showing VGLUT1- or VGAT-positive glutamatergic or GABAergic neurons in UCOs at day 136. Insets show magnified views of the indicated neurons. Arrows indicate the colocalization of VGLUT1 and VGAT in the neuronal axon region. Arrowheads indicate VGAT puncta on the MAP2-positive axon. (M) Immunofluorescence images showing oligodendrocyte lineage marker expression (OLIG2, NG2, and MBP) on the edge of UCOs at day 136. (N) Immunofluorescence images showing a full section of UCOs/SA organoids marked with the anti-C-CASP3 antibody at day 136. Bar graph represents the percentages of C-CASP3-positive cell populations in UCOs/SA organoids at day 136. Quantitative data from immunofluorescence images are expressed as the mean ± SEM. *P < 0.05, **P < 0.01, ***P < 0.001, and ns: not significant. Scale bars, 200 μm (G and M), 50 μm (L), 10 μm (L; magnified image), and 1 mm (N).

Previous studies have demonstrated that brain organoids precisely recapitulate human brain development with various types of neurons and nonneuronal cells, like the fetal brain [1,2]. To validate whether UCOs also represent cortical brain development, we analyzed the total transcriptomes of UCOs at days 40, 64, and 120 and compared these with SA organoids. Although the UCO samples were randomly selected, the high-quality SA samples were selected from a heterogeneous SA organoid population according to the QC-yield_10_ criteria for both diameter and FOXG1-mCherry intensity. The UCOs and SA organoids exhibited similar transcriptome features at each time point, showing high values in the correlation matrix and similar distributions in the PCA plot (Fig. 3A and B). Intriguingly, time-dependent differences in the transcriptional profiles were rarely observed in either organoid until day 64; however, by day 120, distinct patterns of gene expression emerged. This indicated that extensive transcriptional changes occurred at specific time points during cerebral development. Furthermore, we found prominent expression of NPC-specific genes at day 40, followed by a decrease in expression at days 64 and 120 (Fig. 3C). Expression of *TUBB3*, an early neuronal marker, was substantially increased at days 40 and 64 but decreased by day 120 (Fig. 3D). By contrast, *MAP2*, a marker indicative of more mature neurons than those detected by *TUBB3*, was highly expressed at day 120. Following the expression of intermediate progenitor cell (*EOMES*)- and early cortical neuron (*TBR1*)-specific genes at day 64, *BCL11B* and FAM107 showed sequential expression at day 120 when the cortical neurons were actively generated and migrated into specific cortical layers. Gliogenesis had occurred by day 120, and genes associated with oligodendrocyte precursor cells (OPCs) were expressed gradually from day 64 (Fig. 3D). These results indicated that UCOs mimic neuronal differentiation during early brain development by establishing NPCs and generating cortical neurons and glial cells.

We then aligned the transcriptome data of the UCOs and SA organoids with a set of genes that are known to be highly enriched in various brain regions according to transcriptome data from the human fetal brain (stages 4 to 5; brainspan.org; Fig. 3E). We generated gene sets that were specific to each brain region using the top 50 DEGs and manually excluded genes if they also exhibited high expression in other regions. We found a predominant expression of dorsal telencephalic markers in UCOs at day 120, but this was not observed at day 40 or 64. Several markers of exclusively the ventral telencephalon were also expressed in UCOs. However, the transcriptomes of the SA organoids did not correlate with region-specific marker expression, which indicated a lack of specific guidance toward the dorsal telencephalon in the SA group. These findings indicate that the UCOs underwent direct differentiation into the dorsal telencephalon, which became apparent at day 120 following the onset of organoid culture.

We further analyzed the gene expression profiles of organoids and compared them to the transcriptional changes observed during human cortical development in vivo, using the unbiased TMAP assay as reported previously [18,23] (Fig. 3F). We listed the DEGs from the UCOs and SA organoids, conducted normalization using the transcriptomes of the iPSCs, and ranked the DEGs according to their *P* values to perform a quantitative assay based on the rank–rank hypergeometric test [18,24]. We also concurrently ranked the DEGs of the listed developmental periods of the human cortex and compared them with the DEGs of the UCOs and SA organoids using TMAP. Intriguingly, we found that both the SA and UCO organoids exhibited gradual enrichment of DEG patterns from stages 2 to 7 in the fetal brain by day 120; this is consistent with a previous report [23]. It is noteworthy that at day 120, the ranking of UCO DEGs overlaps more closely with those of the human fetal brain than with SA organoids. These results indicated that the UCOs successfully recapitulated the timeline of human fetal brain development.

To explore the generation of diverse neural populations during cortical maturation, we conducted immunohistochemistry of key neuronal markers expressed in organoids (Fig. 3G to M). PAX6, a transcription factor known for eliciting neurogenesis, showed robust expression in NPCs within the neural rosettes of the UCOs at day 40 (Fig. 3G and H). Additionally, TBR2, a marker of intermediate progenitors (IPs) in the dorsal telencephalon, exhibited marked expression at day 56 (Fig. 3G and I). Subsequently, we examined the immunofluorescence signals of cortical layer markers, such as TBR1, CTIP2, and SATB2, at various time points (Fig. 3G and J). The UCOs serially expressed these markers at the periphery of neural rosettes at days 64 and 85, although their expression in the UCOs was lower or delayed compared with that in the SA organoids. These findings indicated that cortical layer specification was slightly delayed in the UCOs compared with the selected SA organoids, which was not observed with TMAP.

Despite this minor delay in the UCOs, a high number of vesicular glutamate transporter 1 (VGLUT1)^+^ glutamatergic neurons were detected at day 105, and vesicular γ-aminobutyric acid (GABA) transporter (VGAT)^+^ GABAergic neurons appeared at day 136 (Fig. 3G and L). The glutamatergic and GABAergic synaptic vesicles coexisted on the neuronal axon, which indicated excitatory–inhibitory synaptic connections in the UCOs at day 136 (Fig. 3L, magnified image). In addition, astrogenesis was induced prominently in the UCOs, as evidenced by a higher S100β-positive cell population in the UCO group than in the SA group (Fig. 3G and K). The expression of OPCs and oligodendrocyte markers, such as OLIG2, NG2, and MBP, indicated that oligodendrogenesis and myelination also occur in UCOs during this period (Fig. 3M).

Although brain organoid models have demonstrated physiological similarities to in vivo brain development, the cultivation of enlarged organoids over prolonged periods results in the formation of a necrotic core due to the absence of the vascular system necessary for supplying nutrients and oxygen [25,26]. Because of the large size of UCOs at day 136 (approximately 4 mm in diameter), we examined the necrosis within the organoids by staining for cleaved caspase-3 (C-CASP3; Fig. 3N). As expected, approximately 30% of C-CASP3^+^ cells were detected in the core of the UCOs, and there was no evidence of CD31^+^ vascular cells generated in the UCOs after day 136 (Fig. S3A). However, despite their larger size, the UCOs showed enhanced cell viability with a markedly lower level of necrosis compared with SA organoids (Fig. 3N). Taken together, we suggest that the UCOs displayed a dorsal telencephalon identity and effectively recapitulated the early stage of human fetal cortical development. Thus, we established UCOs as an appropriate in vitro model that faithfully emulates the features of developing brain tissue.

### Neural networks are well established in UCOs with synaptic transmission of excitatory and inhibitory neurons

**Fig. 4.**
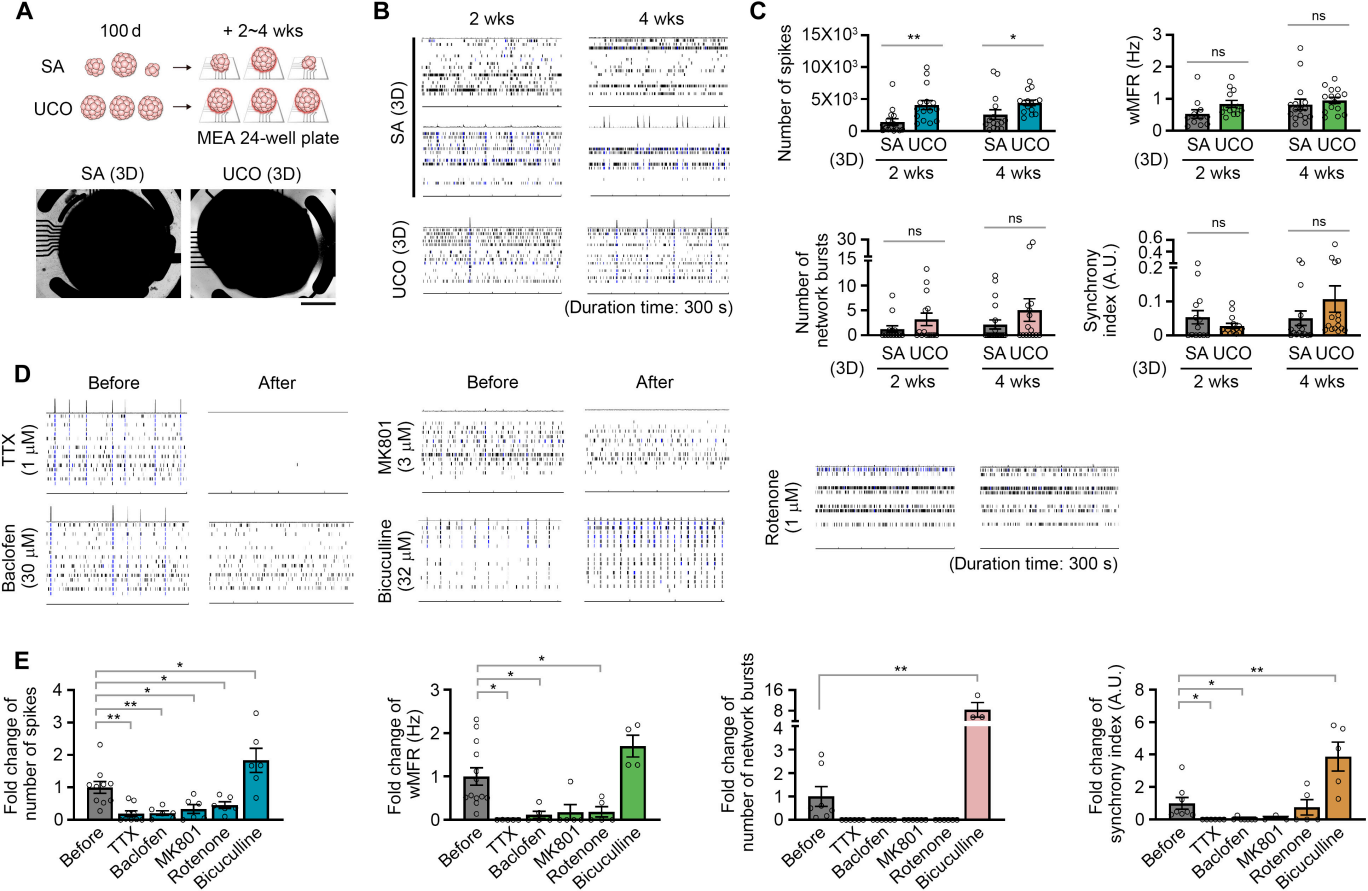
Enhanced neural network signals measured in UCOs with excitatory and inhibitory neuronal responses. (A) Schematic illustration of the procedures to attach UCOs/SA organoids to the 24-well MEA plates from day 100. Bright-field images show the organoids attached to the 16 electrodes of the wells. (B) Representative spike and burst raster plots of the UCOs/SA organoids at 2 and 4 weeks after attachment. Black bars represent spikes. Blue bars that are thicker than the black bars represent mean bursts (i.e., a cluster of spikes). Network bursting is marked with the spike histogram above the raster plots. (C) Bar graphs showing the number of spikes, number of bursts, weighted mean firing rates (wMFRs), and synchrony indices of UCOs/SA organoids at the indicated time points. (D) Raster plots of UCOs at 4 weeks showing the neuroelectrical signals before and after treatment with the indicated drugs. (E) Bar graphs showing the fold changes of the number of spikes, number of bursts, wMFRs, and synchrony indices after drug treatment in UCOs. The total duration of all raster plots was 300 s. Quantitative data from the MEA recording are expressed as mean ± SEM. *P < 0.05, **P < 0.01, ***P < 0.001, and ns: not significant. Scale bar, 1 mm.

We next investigated whether UCOs developed neural networks using a MEA. To examine the effect of organoid uniformity on the reliability of functional outcomes, we randomly selected both UCOs and SA organoids for this experiment. Organoids at day 100 were attached to the MEA 24-well plate and cultured for an additional 4 weeks, with signal recording every 2 weeks (Fig. 4A). Four weeks after organoid attachment, both the UCOs and SA organoids exhibited mature electrophysiological signals, which were characterized by systematic network bursts (Fig. 4B and C). However, in the SA group, only a limited number of organoids showed well-established neural networks. Signal quantification revealed that the majority of UCOs exhibited improved spikes, weighted mean firing rates, and increased network busting and synchrony, although some showed immature signals like those exhibited by the SA group (Fig. 4B and C). These results indicated that UCOs are functionally mature cortical organoids that exhibit systematic neural network signals in a 3D structure.

Next, we investigated whether UCOs respond as expected to various ion-channel blockers and neurotransmitter-binding receptor antagonists/agonists. Treatment of UCOs on MEA plates with TTX, a sodium channel blocker, at 1 μM for 10 min abolished electrical activity (Fig. 4D and E). MK801 (dizocilpine), a noncompetitive NMDA (*N*-methyl-d-aspartate) receptor antagonist that selectively binds to the phencyclidine binding site of glutamatergic neurons, greatly reduced spike numbers and led to the extinction of network activity. Another important measure was the responsiveness of UCOs to GABAergic neuron agonists (baclofen) and antagonists (bicuculline; Fig. 4D and E). These results indicated that the UCOs had established a GABAergic inhibitory neural network that aligned with the immunostaining data at day 136 (Fig. 3L). In addition to the experiments that interrupted neurotransmission, the inhibition of the mitochondrial respiration pathway using rotenone also reduced overall electrical activity (Fig. 4D and E).

Although we confirmed the existence of networking signals in the UCOs, some of them were unresponsive. Because of the technical requirement of ensuring close attachment to the 2D MEA plate for signal recording, the surface area of the organoid available for detection was limited. Therefore, we speculated that the uneven distribution of neurons and glial cells in the UCOs during corticogenesis caused “zones of calm”, which resulted in the unresponsiveness of neuroelectrical signals (Fig. S3B). Indeed, we confirmed substantial differences in the neuroelectrical signals among the 4 pieces of UCOs, despite originating from the same UCO (Fig. S3C). To improve the homogeneity of neuronal signals from the UCOs, we dissociated the UCOs into single cells and attached them to the MEA plate for 2 to 5 weeks (Fig. S4A). We confirmed the gradual maturation of neural network signals: We observed increased network bursting and synchronization at week 5 (Fig. S4B and C). However, the data quality from the 2D neurons derived from the SA organoids was insufficient, which may have been due to the inherent heterogeneity of the SA organoids. These results indicated that UCOs are composed of more mature neurons with higher synaptic activity than SA organoids. In addition, upon pharmacological blockade, UCO-derived 2D neurons displayed perturbations in synaptic transmission and cellular respiratory systems, as expected (Fig. S4D and E). Therefore, we concluded that UCOs are a reliable brain organoid model that exhibit spontaneous neural oscillation signals via excitatory and inhibitory neurotransmission.

### RTT-UCO models show defective neurite outgrowth that can be restored by treatment with a sigma-1 receptor agonist

**Fig. 5.**
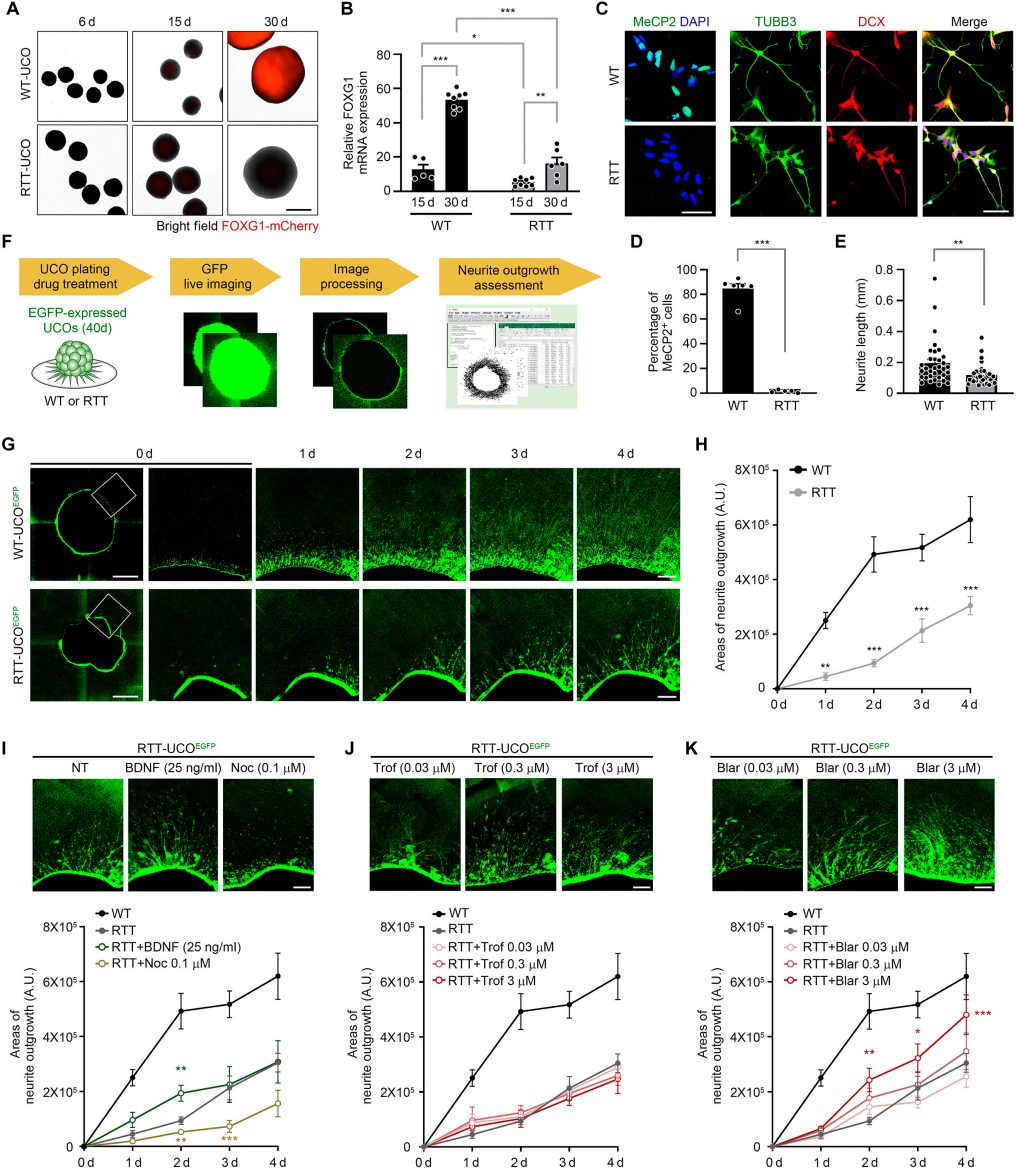
RTT-UCOs exhibited reduced neurite outgrowth of early neurons, which was recovered by treatment with blarcamesine. (A) Live images showing FOXG1-mCherry expression in WT- and RTT-UCOs at the indicated time points. (B) Bar graph of relative FOXG1 mRNA expression in the WT- and RTT-UCOs at days 15 and 30. (C) Immunofluorescence images showing MeCP2 expression in 2D neurons derived from WT- and RTT-UCOs at day 30 and neurite morphology marked with anti-TUBB3 antibody staining. Anti-doublecortin (DCX) antibodies were used to label newly generated neurons. (D and E) Bar graphs of the percentage of MeCP2-positive cell populations (D) and the neurite lengths (E) of 2D neurons derived from WT- and RTT-UCOs. (F) Schematic diagrams illustrating the overall neurite outgrowth assessment process via the live imaging of EGFP-expressed UCOs. (G) Live images showing real-time neurite outgrowth in WT- and RTT-UCOs over 4 d. (H) Neurite outgrowth curves showing gradually increasing lengths of outgrown neurites from 3D organoids. (I to K) Immunofluorescence images at day 4 and neurite outgrowth curves in the indicated conditions (NT, nontreated; Noc, nocodazole; Trof, trofinetide; Blar, blarcamesine). Significance of each group was calculated by comparing with the RTT group at indicated time point. Quantitative data from the fluorescence images are expressed as the mean ± SEM. *P < 0.05, **P < 0.01, and ***P < 0.001. Scale bars, 1 mm (A and G), 50 μm (C), and 100 μm [magnified images in (G) and (I) to (K)].

To assess whether UCOs can also be used as a neurodevelopmental disease model, we applied the UCO culture method to a previously established methyl CpG binding protein 2 (MeCP2) heterozygous-truncated mutant (MeCP2-hTM) model of Rett syndrome (RTT) [27]. We additionally generated FOXG1-mCherry-expressed RTT iPSCs, which were genetically engineered using the CRISPR/Cas9-based knock-in system (Fig. S1A). We found that the RTT-UCOs expressed minimal *FOXG1* mRNA, with low expression of FOXG1-mCherry during the early stages of brain organogenesis, which indicated an association between MeCP2 and the forebrain specification during embryogenesis (Fig. 5A and B). Moreover, compared with WT neurons, the newborn neurons derived from the RTT-UCOs at day 30 showed reduced neurite length and an absence of MeCP2 expression (Fig. 5C to E). These results are consistent with previous reports that showed that loss of MeCP2 function induces the generation of immature neurons that exhibit defective neurite outgrowth [28,29]. In addition to impaired neurite outgrowth, RTT-UCOs showed marked decrease in neuronal activity, as indicated by a marked decrease in network bursts compared to those in WT-UCOs (Fig. S4F). This reduction in neuronal activity suggests that MeCP2 deficiency disrupts synaptic connectivity and network formation, which are essential for effective neurite extension and branching. The diminished electrical activity in RTT-UCOs provides a mechanistic link to the defective neurite outgrowth.

Based on these phenotypes, we established a drug testing platform to evaluate the efficacy of RTT-targeted drugs using live imaging of neurite outgrowth in 3D RTT-UCOs (Fig. 5F). We generated EGFP-labeled UCO models derived from genetically engineered iPSCs to allow for real-time observation of neurite outgrowth from the surface of the UCOs (Fig. S1H). Both the WT and RTT EGFP-UCOs were cultured for 40 d and attached to a well plate for live imaging to monitor their neurite outgrowth over a 4-d period using the HCS system. The acquired raw images were processed and analyzed to quantify the areas of outgrown neurites labeled by EGFP. We confirmed that, similar to the single-neuron cells, both the dendrite length and branch number of the RTT-UCOs were lower than those of the WT UCOs when measured 4 d after the onset of UCO attachment (Fig. 5G and H). However, these phenotypes were not restored by treatment with BDNF, which is known to be notably reduced in RTT patients [30,31] and mouse models [32]. This indicated that short periods of elevated BDNF levels were not sufficient to overcome the loss of MeCP2 function in the neurite outgrowth of early neurons (Fig. 5I). Nocodazole was used as the negative control via the inhibition of microtubule synthesis during neurite growth of neurons. Trofinetide is a Food and Drug Administration-approved synthetic insulin-like growth factor 1 analog that enhances BDNF effects by indirectly activating the receptor signaling cascade [33,34]. When we tested trofinetide in the established model, neurite outgrowth was largely unaffected in 40-d-old RTT-UCOs (Fig. 5J). By contrast, blarcamesine, which is another RTT-targeted drug that is currently undergoing phase 3 clinical trials, sequentially increased neurite lengths in a dose-dependent manner (Fig. 5K). Blarcamesine is a sigma-1 receptor (SGMAR1) agonist that has been developed for the treatment of Alzheimer’s disease and RTT [35]. It is noteworthy that the activation of SGMAR1 stimulates the neurite outgrowth of cortical neurons and increases the spine length of pyramidal neurons in the hippocampus [36,37]. Taken together, our results suggest that an SGMAR1 agonist stimulates neurite growth in 3D RTT-UCOs during short periods of drug treatment. In conclusion, RTT-UCOs may be used to assess drug efficacy during the early stage of human brain development; moreover, they may provide an HTS platform based on the mechanisms underlying the pathogenesis of RTT.

## Discussion

Over the past decade, remarkable advances in cerebral organoid culture have enabled direct exploration of brain development and the pathogenesis of neurological disorders in laboratory culture dishes. However, the considerable variability in generation protocols, quality, and productivity of cerebral organoids among laboratories challenges their reliability as a platform to evaluate drug efficacy. To facilitate the broad application of cerebral organoids in HTS platforms, we must establish a standardized protocol that ensures intra-batch homogeneity and batch-to-batch reproducibility within generations. Here, we provide a reliable protocol for establishing a high-quality cerebral organoid model with standardized morphology that is suitable for the HTS platform.

Our optimized protocol integrates several critical components that ensure the uniformity, high yield, and reproducibility of cerebral organoids, supported by rigorous quantitative parameters for accurate assessment (Table S4). The microwell system contributed to controlling the initial EB size and ensuring consistency in EB formation, similar to other protocols using AggreWell [38]. However, the primary factors driving reproducibility and differentiation efficiency stem from broader protocol optimizations, including the strategic use of iPSC clumps, the refinement of growth factors, and the integration of the FOXG1-mCherry reporter system for real-time monitoring of telencephalic differentiation.

We generated UCOs from clumped iPSC colonies and avoided the harsh detachment of individual cells from the colonies. Typically, the uniform size of brain organoids is achieved by controlling the seeding density of single embryonic stem cells (ESCs) or iPSCs via enzymatic dissociation of stem cell colonies from 2D culture dishes [1,15]. However, such procedures are detrimental to stem cells because of the damage inflicted on surface proteins essential for intercellular communication and the reduction in cell viability due to abnormal karyotypes [39,40]. Although many studies have successfully used single-cell dissociation and long-term ROCK inhibition strategies to establish brain organoid models, in our system, singularized iPSCs rarely differentiated into dorsal forebrain cells, which is in contrast to the clumped colony-derived organoid model. It seems that the iPSCs in our system are likely to be more sensitive to shear stress during the cell-dissociation process and to long-term ROCK inhibition due to the inherent diversity among iPSC lines [41,42]. By confining the iPSC colonies of various sizes to microwells of uniform dimensions, we effectively resolved the issue of heterogeneous EB sizes resulting from the SA of iPSC clumps. This technical approach allows for the enhancement of the differentiation efficiency of iPSCs that are vulnerable to mechanical and chemical stress.

Using a microwell system with 400-, 600-, and 1,000-μm well diameters, we facilitated the reproducible formation of single EBs per well. Our findings indicate that a well diameter of 1,000 μm is optimal for telencephalic differentiation, providing a balance between cell–cell interaction and signaling gradient that enhances differentiation efficiency (Tables S3 and S4). Recent studies have demonstrated that smaller EBs (below 500 μm) exhibit reduced neuroepithelial bud formation and fail to form organoids [43], while large EBs contain more neural precursor cells, enhancing neural lineage differentiation [44]. Thus, using the 1,000-μm microwell in our system supports the production of high-yield UCOs with optimized telencephalic differentiation.

Despite optimizing EB size, initial tests indicated the need for additional factors to enhance telencephalon-specific differentiation. We refined our protocol by optimizing growth factors and supplements, including Wnt inhibitors, and utilized the FOXG1-mCherry reporter system for real-time monitoring of differentiation, enabling timely adjustments to culture conditions (Fig. 2). Wnt inhibition, combined with dual SMAD inhibition, has improved the homogeneity of neural stem cell identities, specifically directing precise commitment to the telencephalic lineage [45], and we similarly confirmed its enhancing effect on FOXG1 expression. Furthermore, we observed that the synchronization of EB growth rates was driven by Wnt inhibition during neural induction. It is known that the Wnt protein acts as a mitogen of cycling cells, and inhibition of the Wnt pathway causes the synchronization of a cell cycle [46]. Therefore, Wnt inhibition accelerated the homogeneous growth of EBs while concurrently promoting the achievement of a uniform telencephalon identity in our system.

In this context, the FOXG1-mCherry reporter system allowed us to track the progression of telencephalic differentiation in real time, providing valuable feedback to fine-tune culture conditions (Fig. 2). By adjusting growth factors and supplements based on FOXG1 expression levels, we greatly improved the homogeneity of neural stem cell populations and precisely directed cells toward the cortical lineage (Fig. 3). This system also facilitated organoid selection, allowing us to align size-based selection criteria with differentiation status, which ultimately enhanced the reliability and reproducibility of organoid generation (Table S4). By integrating both Wnt inhibition and the real-time tracking system, our protocol not only accelerated uniform EB growth but also promoted consistent telencephalic differentiation.

Our systematic comparison of whole transcriptome data from UCOs using a human fetal brain database demonstrated that spatiotemporally regulated fate determination is established in UCOs, much like the in vivo system. It is notable that randomly selected UCO samples exhibited a similar distribution in the global gene expression profile as carefully selected SA organoid samples. However, the expression of cortical layer markers was lower in the UCOs than in the SA organoids, which was indicative of inherent differences between UCOs and SA organoids that could not be discerned in the heatmap or TMAP. We speculate that the prolonged neural induction period of UCOs compared with that of the SA protocol affected the progression of subsequent stages. Indeed, the extended duration of neuroectodermal differentiation can affect the course of NPC proliferation and differentiation [47]. Nevertheless, the UCOs successfully exhibited glutamatergic and GABAergic neurogenesis, alongside nonneuronal cell types, such as astrocytes and oligodendrocytes in the later phases of organoid development. Thus, the delayed corticogenesis during the early stages was partially compensated for as organoid development progressed. Moreover, the UCOs showed advanced neuronal functionality with considerable responsiveness to various neurotransmission blockers. The neuroelectrical activity and networking of 2D neurons derived from the UCOs were superior to those of the SA organoids, which indicated that the UCOs retained highly functional neurons and generated a mature neural network system. These physiological advantages support the notion that UCOs can serve as a valid system for researching neurotransmission and neural networks in developmental brain tissue as well as neuronal dysfunction in neurodevelopmental disease models.

Accordingly, we established RTT-UCOs using MeCP2-mutated iPSCs and confirmed neurite outgrowth alterations in early-born neurons. Based on this phenotype, we expanded our model into a live imaging platform coupled with an HCS analysis system. It is notable that neither the elevation of BDNF levels nor treatment with trofinetide recovered the defective neurite outgrowth in our system. Therefore, RTT-UCOs in the early developmental phase do not respond to such treatments under the indicated conditions; alternatively, the duration of drug treatment may not have been sufficient to demonstrate the efficacy of trofinetide and BDNF. However, blarcamesine, another RTT-targeted drug, dose-dependently rescued this phenotype, which demonstrates its acute efficacy in the early developmental phase of cerebral organoids. These results suggest that our system can facilitate target-based drug efficacy analyses across distinct developmental stages and treatment durations.

However, our UCO model has several limitations that require refinement to achieve the status of an ideal 3D-biomimetic model (Table S4). First, the UCOs exhibited the neuroectodermal lineage of cells but lacked the endothelial blood vessel cells; moreover, similar to other cerebral organoid models, few microglia differentiated from the mesodermal lineage [2]. Although our systematic culture processes for UCOs could improve the viability of core cells until later stages of organoid culture, they were not sufficient to emulate the internal in vivo microenvironment for providing adequate nutrients and removing inappropriate metabolites. Second, the absence of extracellular matrix (ECM)-mediated signaling transduction in our system hinders the ability to explore the radial migration of newborn neurons and the pathogenesis of related diseases, such as microgyria and lissencephaly [48,49]. Other strategies, such as combining coculture systems for different lineages of cells and integrating ECM-like materials, need to be considered to develop UCOs into a more reliable cerebral organoid model.

Additionally, our functional assays, primarily optimized for 2D-based systems, may not fully capture the complex 3D neural networks within organoids. Measuring electrophysiological signals from 3D organoids on a 2D MEA platform poses challenges due to the labor-intensive steps required for organoid adhesion and the limited scope of electrical activity observation. Despite the successful generation of uniform organoids, the spatial heterogeneity of neural populations within the organoid restricts signal recording to specific regions of the spherical structure. Recent advances in bioelectronic platforms, such as flexible 3D electrodes that surround organoid surfaces and multichannel neural probes that measure neural activity within the organoid, offer promise for improving the ability to detect neural signals in our model [50].

While the 2D approach has limitations in observing axonal growth and synaptic maturation 3-dimensionally (Fig. 5), it provides valuable quantitative data that are particularly useful for drug screening applications. The ability to generate reproducible and consistent data from 2D assays facilitates high-throughput analysis and allows for real-time monitoring of drug responses, which is critical in pharmacological studies. Given our goal of developing UCOs for drug efficacy and safety screening, the 2D-based evaluation system remains a practical and effective approach. However, to fully capture the intricacies of 3D cellular interactions and neural network dynamics, future innovations in 3D analytical methods, such as advanced imaging techniques and bioelectronic interfaces, will be essential. These developments will further refine our model’s ability to mimic in vivo conditions and enhance its applicability in neurological research and therapeutic development.

In conclusion, our enhanced method for manufacturing high-quality UCOs represents a substantial improvement upon existing protocols that typically yield heterogeneous brain organoids. Our improved approach offers new avenues for establishing an optimal brain organoid system for screening both drug neurotoxicity and efficacy. We anticipate that our UCO method will offer valuable insight into drug development for neurological disease models, as exemplified by its applicability to the RTT model.

## Data Availability

All relevant data are available from the corresponding author upon reasonable request.
