# Supplementary material for: Manufacturing Uniform Cerebral Organoids for Neurological Disease Modeling and Drug Evaluation
Source: Biomater Res. 2024 Nov 6;28:0104. doi: 10.34133/bmr.0104 (PMC11538552; doi:10.34133/bmr.0104)
Supplement: Supplementary 1 — Figs. S1 to S4 Tables S1 to S4 [file bmr.0104.f1.zip › Supplementary figures.docx]

**Supplementary figure legends**

**Fig. S1. Protocols for the cerebral organoid culture, validation data of cell lines, and the results of additive organoid models.** (A) Schematic illustration of the target exon region of *FOXG1*, the CRISPR/Cas9-based knock-in system, and the DNA gel-running results showing the edited cell lines that contained the mCherry expression cassette after the last 3’ exon of *FOXG1*. (B) Schematic diagrams illustrating the three procedures for generating cerebral organoids. (C) Graph showing the correlation between organoid diameter and FOXG1-mCherry intensity in SA organoids at day 30. (D) Bright-field images showing the EBs of the 96W (2 × 10^4^ cells/well) group at the indicated time points. (E–G) Dot plots showing the organoid diameters (E), mCherry intensity (F), and bar graph showing the relative *FOXG1* mRNA expression (G) of the two 96W groups (0.9 × 10^4^ cells/well and 2 × 10^4^ cells/well) at days 15 and 30. (H) Illustrations of the target sites for the CRISPR/Cas9-based EGFP knock-in system. Gel-running images show the results of the genetically engineered cell lines containing the EGFP-expression cassette. All quantitative data are expressed as mean ± standard error of the mean (SEM), and significance of each group was calculated by comparing with the SA group. **P* < 0.05, ***P* < 0.01, ****P* < 0.001, and ns: not significant. Scale bars, 1 mm.

**Fig. S2. Stimulation of FOXG1 expression by the Wnt inhibitor.** (A) Live images showing the FOXG1-mCherry-expressed organoids in the indicated groups at each time point. (B–D) Dot plots representing the organoid diameters (B), mCherry intensity (C), and bar graph showing the relative *FOXG1* mRNA expression (D) of all groups at the indicated time points. (E and F) Tables showing the CV values of organoid diameter (E) and mCherry intensity (F) in the indicated conditions. (G) The QC-yield_10_ of organoids calculated from organoid diameter and mCherry intensity (day 30). (H and I) Dot plot showing the mCherry intensity (H) and bar graph presenting relative *FOXG1* mRNA expression (I), normalized by its expression in iPSCs, in the organoids of each group at day 30. (J) Immunofluorescence images showing a cross-section of all groups of organoids stained with anti-SOX2 and anti-MAP2 antibodies at day 30. The nucleus was marked with DAPI. (K) Dot plots showing the rosette thickness of organoids in the indicated groups. All quantitative data are expressed as mean ± standard error of the mean (SEM), and significance of each group was calculated by comparing with the SA group. **P* < 0.05, ***P* < 0.01, ****P* < 0.001, and ns: not significant. Scale bars, 1 mm.

**Fig. S3. Heterogeneous morphology and physiology of each UCO.** (A) Immunofluorescence images showing anti-CD31 and anti-MAP2 antibody staining of UCOs at day 136. The nucleus was marked with DAPI. (B) Immunofluorescence images showing the distribution of neurons (MAP2) and glial cells (GFAP and S100β) in UCOs at day 136. Insets show the magnified views of the indicated organoid regions. (C) Schematic diagrams of the procedure for dissecting the UCOs into four pieces and attaching them to MEA plates. Raster plots showing the neuroelectrical signals of the four organoid pieces over 300 seconds. Scale bars, 100 μm (A), 1 mm for the full organoid images in (B), and 200 μm for the magnified views in (B).

**Fig. S4. Electrophysiological recordings of 2D-neurons derived from UCO and SA organoids.** (A) Schematic illustration of the seeding procedure of the 2D cells derived from 3D organoids and the incubation timeline for detecting mature neural network signals. Bright-field images show the seeded cells from the UCOs and SA organoids on the MEA plates. (B) Representative raster plots of the 2D cells from the 3D organoids at the indicated time points from the onset of cell seeding. (C) Bar graphs showing the number of spikes, number of bursts, wMFRs, and synchrony indices from the 2D cells at two time points. (D) Raster plots of the 2D cells from the UCOs at 5 weeks showing neuroelectrical signals before and after treatment with the indicated drugs. (E) Bar graphs showing the fold changes of the number of spikes, number of bursts, wMFRs, and synchrony indices after drug treatment in the 2D cells from the UCOs 5 weeks after cell seeding. (F) Bright-field images, raster plots, and bar graphs showing electrophysiological recording data of WT- and RTT-UCOs at day 120. Scale bar, 500 μm.
