# Supplementary material for: Manufacturing Uniform Cerebral Organoids for Neurological Disease Modeling and Drug Evaluation
Source: Biomater Res. 2024 Nov 6;28:0104. doi: 10.34133/bmr.0104 (PMC11538552; doi:10.34133/bmr.0104)
Supplement: Supplementary 1 — Figs. S1 to S4 Tables S1 to S4 [file bmr.0104.f1.zip › Table S1_S2_S3_S4 (1).docx]

**Table S1. List of primers used for the genotyping for targeted integration detection**

| Target site | Sequences (5’-3’) |
| --- | --- |
| *FOXG1* 5’-Junction forward (*FOXG1* locus detection) | ttcctgtccctgcaccac |
| *FOXG1* 5’-Junction reverse (mCherry detection) | cttggtcaccttcagcttgg |
| *FOXG1* 3’-Junction forward (PuroR cassette detection) | gcctgaagaacgagatcagc |
| *FOXG1* 3’ -Junction reverse (*FOXG1* locus detection) | caataaatggccaaaataat |
| *AAVS1* 5’-Junction forward (*AAVS1* locus detection) | TCCTGAGTCCGGACCACTTT |
| *AAVS1* 5’-Junction reverse (PuroR detection) | GTGGGCTTGTACTCGGTCAT |
| *AAVS1* 3’- Junction forward (EGFP detection) | CATGGTCCTGCTGGAGT CGTG |
| *AAVS1* 3’- Junction reverse (*AAVS1* locus detection) | CCTGGGATACCCCGAAGAGT |

**Table S2. List of antibodies used for immunofluorescence staining**

| Antibody | Source | Identifier |
| --- | --- | --- |
| SOX2 | Abcam | ab92494 |
| MAP2 | Abcam | ab11267 |
| PAX6 | Abcam | ab78545 |
| TBR2 | Millipore | AB15894 |
| TBR1 | Abcam | ab31940 |
| CTIP2 | Abcam | ab18465 |
| SATB2 | Abcam | ab51502 |
| VGLUT1 | Synaptic Systems | 135-302 |
| GAD1 | Merck | MAB5406 |
| GFAP | Invitrogen | PA1-10004 |
| S100β | Synaptic Systems | 287003 |
| VGAT | Synaptic systems | 131004 |
| OLIG2 | Abcam | ab109186 |
| NG2 | Abcam | ab129051 |
| MBP | Invitrogen | PA5-78397 |
| Cleaved Caspase 3 | Cell signaling | 9661 |
| TUBB3 | BioLegend | 802001 |
| DCX | Abcam | ab135349 |
| CD31 | Abcam | ab28364 |

**Table S3. Productivity of organoid culture groups**

| **Organoid culture groups** | | **SA** | **UCO** |
| --- | --- | --- | --- |
| Scales of iPSCs^1^ | | 1 plates | 1 plates |
| Number of starting EBs (apprx.) | | 250 EBs  (100%) | 295 EBs  (100%) |
| Number of organoids at day30^2^  (apprx.) (A) | | 190 organoids  (76%) | 260 organoids  (88.1%) |
| QC-yield_10_ (%)^3^ | Diameter  (B) | 32.1% | 100% |
|  | mCherry intensity  (C) | 3.7% | 100% |
| QC-yield_30_ (%)^4^ | Diameter  (B) | 70.4% | 100% |
|  | mCherry intensity  (C) | 14.8% | 100% |
| Number of QC-passed organoids^5^  (AX(B∩C)) | QC yield_10_ | 4.6 organoids | 260 organoids |
|  | QC yield_30_ | 18.5 organoids | 260 organoids |
| **Productivity (%)** | **QC yield_10_** | **1.8%** | **88.1%** |
|  | **QC yield_30_** | **7.4%** | **88.1%** |

^1^iPSCs were cultured in 6-well plates, with each well reaching approximately 80% confluency before generating EBs.

^2^Technically and spontaneously eliminated EBs due to being washed out by their extremely small size or undergoing apoptotic/necrotic cell death, resulting in failure of EB formation.

^3^QC yield (mean±10%) and ^4^QC yield (mean±30%) of day 30 organoids.

^5^Number of organoids which satisfied the criteria of organoid quality control (both organoid diameters within mean ± 10% or 30%, and mCherry intensity within mean ± 10% or 30%).

**
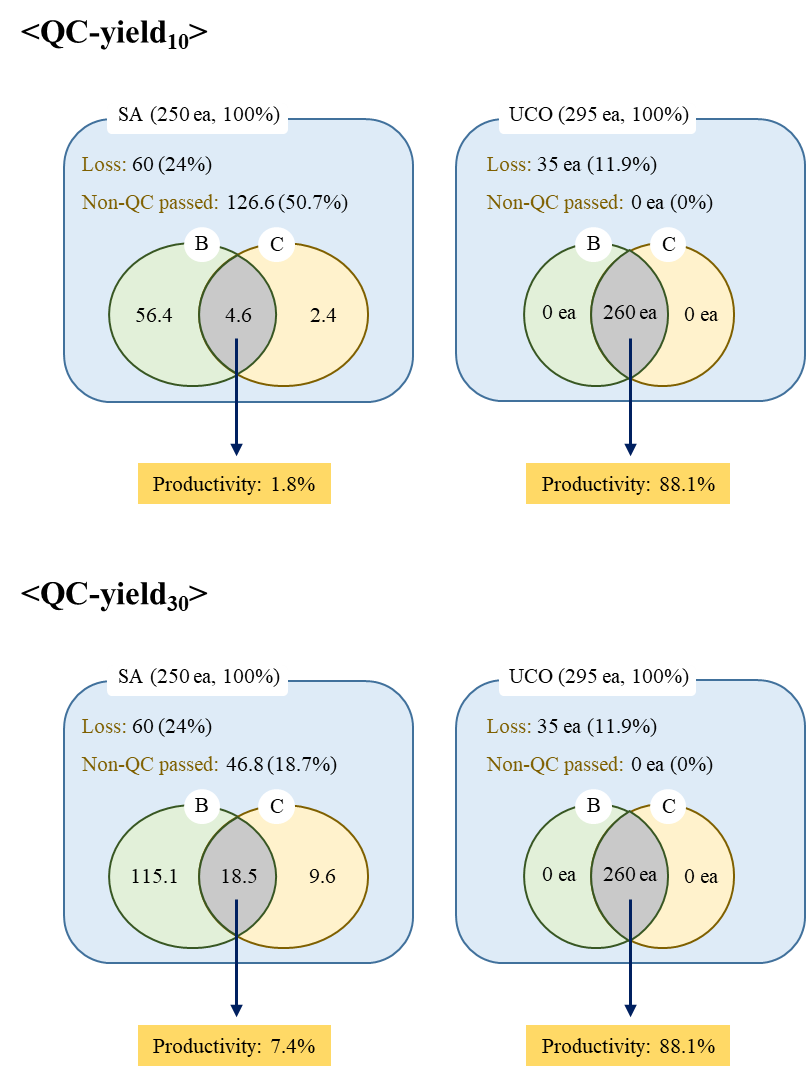
**

<Number of organoids of each group in Table S3 and productivity>

**Table S4. Comparison of UCO protocol with other existed cortical brain organoid methods**

| **Protocols** | | **UCOs** | **Lancaster et al., 2013/2014** | **SA**  **(Pasca et al., 2015)** | **SJ Yoon et al., 2019** | **Sivitilli AA et al., 2020** | **96W**  **(Xiang et al., 2017)** |
| --- | --- | --- | --- | --- | --- | --- | --- |
| Details of procedures | hPSC type | iPSC | ESC, iPSC | iPSC | iPSC | ESC | ESC, iPSC |
|  | Cell dissociation | iPSC clumps | Single cell | iPSC colonies | Single cell | Single cell | Single cell |
|  | EB formation | Microwell  (1000 μm) | U-bottom 96-well plate  (4500 cells/well) | Self- aggregation | AggreWell 800 | V-bottom  96-well plate (12000 cells/well) or AggreWell 800 | U-bottom 96-well plate  (9000 cells/well) |
|  | Neural induction | dSMADi  WNTi | N2 supplement  heparin | dSMADi | dSMADi  WNTi | N2 supplement  heparin | dSMADi  WNTi |
|  | Matrigel embedding | x | o | x | x | o | x |
| **Size uniformity**  **(yield)** | **Original data** | Day30,  QC-yield10, **100%** | Not  measured | Not  measured | Not  measured | **~80%**, AggreWell800; **~100%**, V-bottom plate) | Not  measured |
|  | **In-house data** |  | Not  measured | Day 30, QC-yield10, **32.1**% | Not  measured | Not  measured | Day 30, QC-yield10, **52.9%** |
| **Cerebral differentiation**  **(Quantitative)** | **Original data** | FOXG1 expression  (Day30, QC-yield10, **100%** yield),  Cortical layer marker expression  (Day 64, 85) | Not  measured | Cortical layer marker expression  (Day 52, 76, 137) | >100d differentiation success rate (**80~100%**)  , FOXG1 expression  (Day 25) | Not  measured | FOXG1, PAX6 expression  (Day 21, **~100%** yield) |
|  | In-house data |  | Not  measured | FOXG1 expression  (Day 30 QC-yield10, 3.7%) | Not  measured | Not  measured | FOXG1 expression  (not estimated) |
| Organoid  Functionality analysis | | Electrophysiology analysis | Calcium  live imaging | Calcium  live imaging,  Electrophysiology analysis | Not  shown | Electrophysiology analysis | Calcium  live imaging,  Electrophysiology analysis,  interneuron migration |
| Applications | | RTT-UCO model & drug testing platform | Microcephaly organoid model | Not  shown | Not  shown | Not  shown | Not  shown |
| Strengths | | - High uniformity and reproducibility - Optimized differentiation into telencephalic lineages - Real-time monitoring of differentiation status - Improved productivity for drug screening applications | - Minimal use of exogenous patterning factors - Robust generation of human-specific brain structures - Application for disease modeling | - Simplicity and reproducibility - Generation of laminated cortical structure - Functional maturation of neurons and astrocytes | - High reliability and consistency - Directed differentiation to cortical lineages - Advanced molecular characterization | - High reproducibility and uniformity - Advanced characterization techniques - Self-patterned organoids | - Modeling human interneuron migration - Generation of region-specific organoids - Functional maturation of neurons |
| Limitations | | - 2D-based analysis - Lack of vascularization - Absence of ECM-mediated signals | - Absence of quantitative analysis for uniformity of organoids - 2D-based analysis - Lack of circulation system | - Absence of Quantitative analysis for uniformity of organoids - 2D-based analysis - Absence of ECM-mediated signals - Lack of vascularization | - 2D-based analysis - Lack of vascularization - Absence of ECM-mediated signals - Lack of functional characterization | - 2D-based analysis - Lack of vascularization - Technical complexity | - Absence of quantitative analysis for uniformity of organoids - Lack of vascularization - Absence of ECM-mediated signals |
