# Supplementary figures and images for: Manufacturing Uniform Cerebral Organoids for Neurological Disease Modeling and Drug Evaluation
Source: Biomater Res. 2024 Nov 6;28:0104. doi: 10.34133/bmr.0104 (PMC11538552; doi:10.34133/bmr.0104)

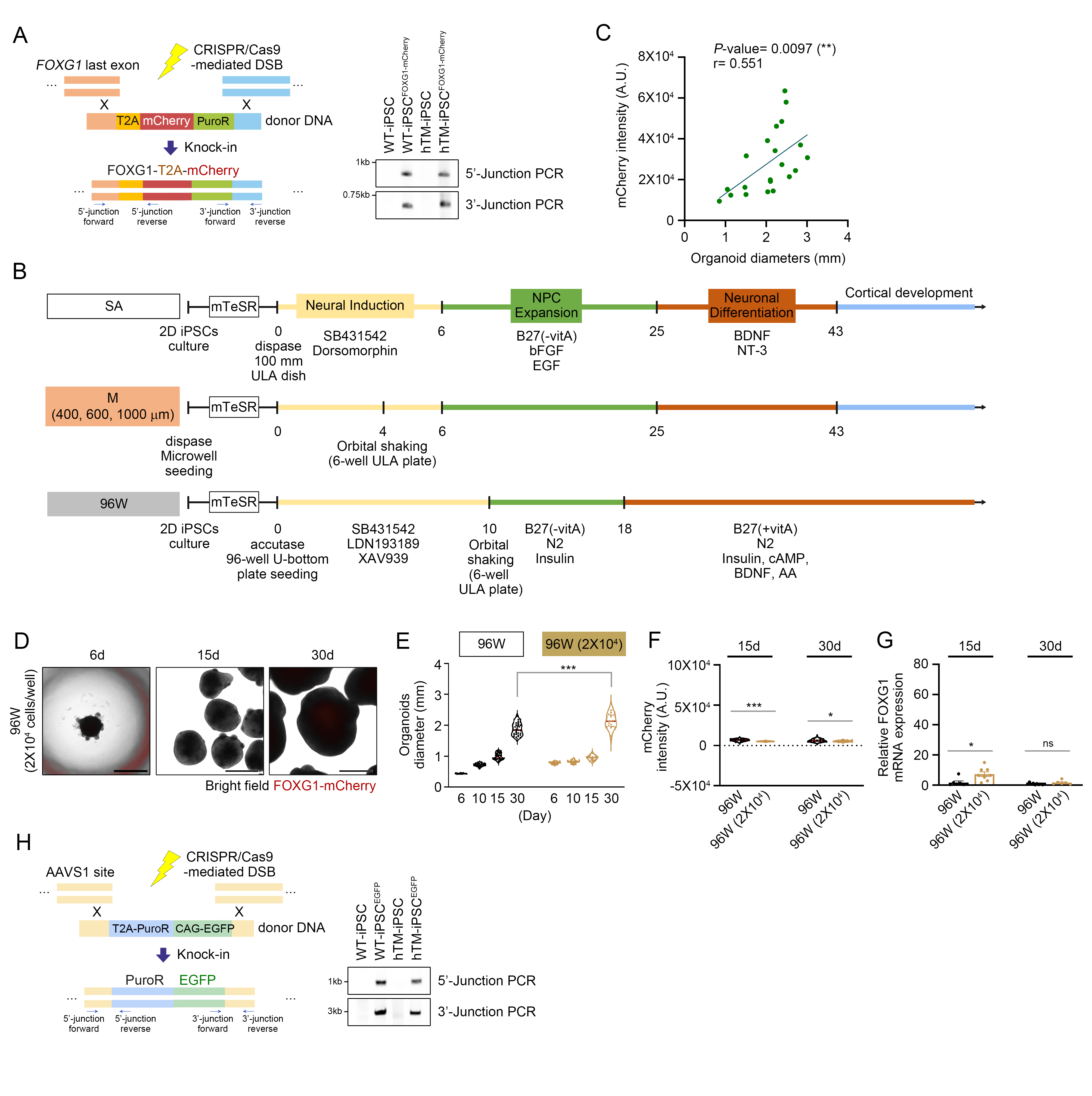

Supplement: Supplementary 1 — Figs. S1 to S4 Tables S1 to S4 [file bmr.0104.f1.zip › Fig. S1.tif]

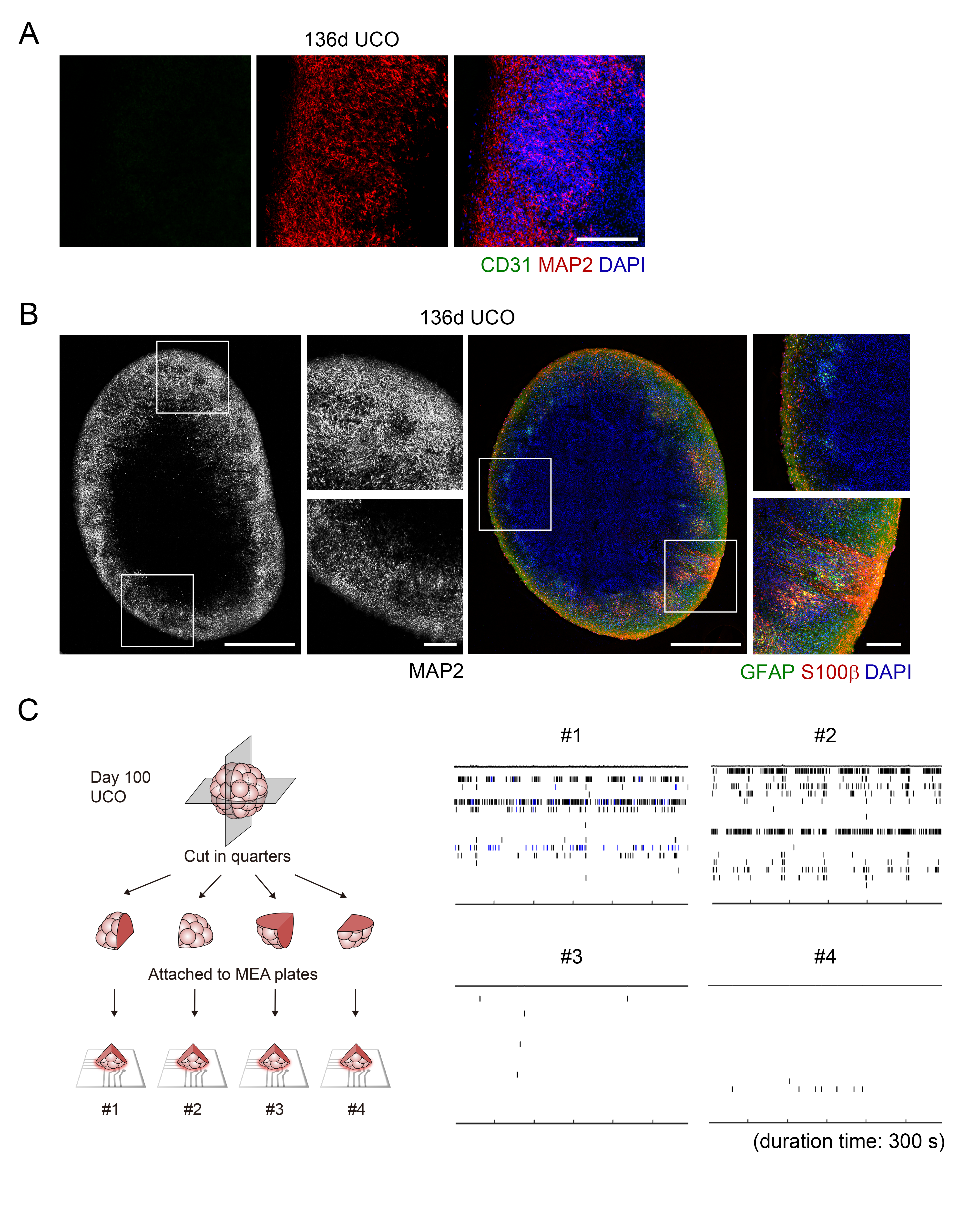

Supplement: Supplementary 1 — Figs. S1 to S4 Tables S1 to S4 [file bmr.0104.f1.zip › Fig. S3.tif]

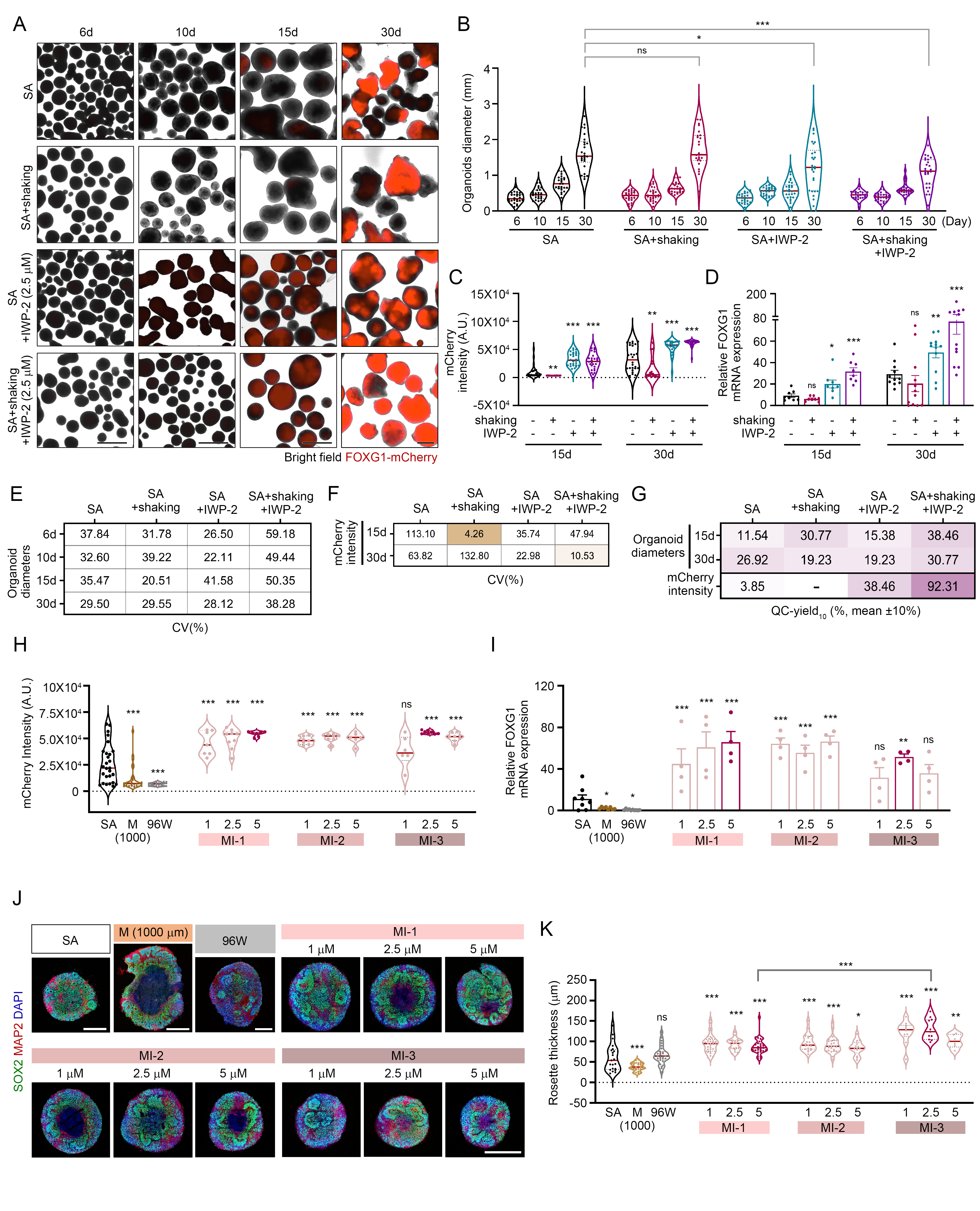

Supplement: Supplementary 1 — Figs. S1 to S4 Tables S1 to S4 [file bmr.0104.f1.zip › Revised Fig. S2.tif]

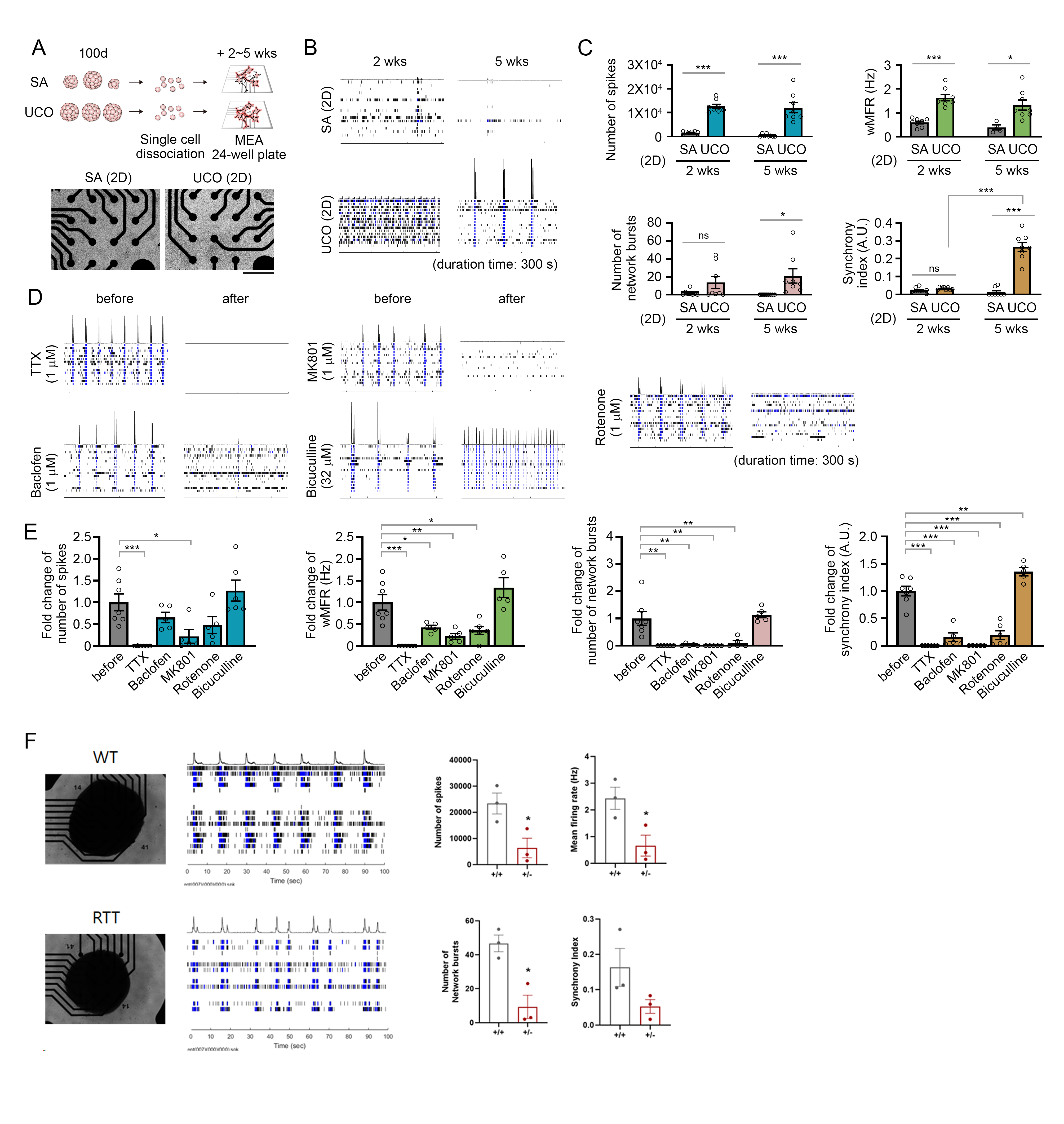

Supplement: Supplementary 1 — Figs. S1 to S4 Tables S1 to S4 [file bmr.0104.f1.zip › Revised Fig. S4.tif]
